# Pharmacologically Induced Accommodation Palsy and the Bioelectrical Activity of the Muscular System: A Preliminary Investigation

**DOI:** 10.3390/diagnostics14090961

**Published:** 2024-05-04

**Authors:** Grzegorz Zieliński, Beata Pająk-Zielińska, Anna Woźniak, Michał Ginszt, Nicola Marchili, Piotr Gawda, Robert Rejdak

**Affiliations:** 1 Department of Sports Medicine, Medical University of Lublin, 20-093 Lublin, Poland; 2 Interdisciplinary Scientific Group of Sports Medicine, Department of Sports Medicine, Medical University of Lublin, 20-093 Lublin, Poland; 3 Department of General and Pediatric Ophthalmology, Medical University of Lublin, 20-093 Lublin, Poland; anna.wozniak@umlub.pl (A.W.);; 4 Department of Rehabilitation and Physiotherapy, Medical University of Lublin, 20-093 Lublin, Poland; michal.ginszt@umlub.pl; 5 Private Practice in Rieti, 02100 Rieti, Italy; nicolajuve89@gmail.com

**Keywords:** myopia, refractive error, vision, muscular system, muscles, sEMG

## Abstract

The aim of this study was to pharmacologically induce accommodative paralysis and evaluate its effects on the bioelectrical activity of the muscular system. The study included two participant groups: those with myopia and those with normal vision (emmetropes). Electromyographic assessments were performed using the Noraxon Ultium DTS 8-K MR 3 myo Muscle Master Edition system. The muscles analyzed in this study were the temporalis, masseter, sternocleidomastoid, trapezius, abdominal muscles, biceps brachii, and the external oblique muscles of the abdomen. It is important to acknowledge that, based on the current findings, it cannot be definitively stated that the observed effects have clinical significance, and additional studies are encouraged.

## 1. Introduction

Myopia is a global social problem. Statistical projections suggest an increase in myopia over the coming years [1]. The etiology of this refractive error is not fully understood. Myopia is associated with the focusing of light rays in front of the retina. It is usually associated with axial elongation of the eyeball. The myopia in question is divided into low myopia of up to −6.00 diopters and high myopia of more than −6.00 diopters (D) [2]. People with myopia are at a higher risk of developing cataracts, glaucoma, macular degeneration, and retinal detachment. Visual problems, especially in uncorrected patients, may be associated with headaches [3,4]. The estimated global prevalence of headaches is 52%, of which 26.0% are associated with abnormal muscle activity and so-called tension-type headaches (TTHs) [5]. Although the etiology of TTH is still unclear, it seems like central synthesis may be involved in the transformation from episodic to chronic TTHs [6].

The connections between the visual system and the stomatognathic system are observable both in research and clinically [7,8]. During a change in visual stimulus (closing eyes) compared to looking straight ahead (without correction of refractive error), there are changes in the bioelectrical activity of the masticatory muscles [7,8]. In addition to the change in function (change in bioelectrical activity), changes within the muscle structures related to the refractive error are observable [9,10].

The smallest unit of the neural control of muscle contraction is the motor unit. The motor unit consists of the alpha motoneuron of the anterior horn of the spinal cord, its axon and all the muscle fibers innervated by this neuron [11]. The central nervous system is responsible for the orderly activation of motor neurons [12]. This is followed by changes in the excitability of the muscle membranes. The electromyographic signal is generated by action potentials resulting from depolarization and repolarization processes [13].

Postural control in the human body is a multifaceted process that involves several neural pathways beyond the pyramidal tract [14]. While the pyramidal tract, which includes the corticospinal and corticobulbar tracts, is crucial for the control of voluntary movements, including those of the face, head, neck (via the corticobulbar tract), and the rest of the body’s muscles (via the corticospinal tract), it is not the primary system responsible for postural control. Instead, postural stability and balance are predominantly managed by the extrapyramidal system, which consists of motor control pathways such as the vestibulospinal tract, reticulospinal tract, and the tectospinal tract [14]. These pathways are integral for maintaining posture and balance, coordinating automatic movements, and reacting to external stimuli to adjust the body’s position [15].

In the corticobulbar tract, axons transmit signals towards muscles innervated by cranial nerves, facilitating movements of the face, head, and neck. The reticular formation (RF), a part of the brainstem, plays a key role in motion control and coordination. It contains functional groups of cells crucial for controlling movements of the eyes, head, and eyelids. Located throughout the brainstem without clear boundaries, the RF is characterized by its extensive network of interconnected nuclei and nerve fibers, allowing for the integration of various sensory inputs and motor responses [16]. This diffuse structure ensures that a cell within the RF can respond to stimuli from multiple receptors, illustrating the complexity of neural integration required for coordinated movement and postural control.

The reticular formation’s location in the midbrain and its dense network of connections highlight its significance in the neural control system, facilitating the integration of visual, vestibular, and somatosensory information essential for maintaining balance and posture. Thus, the connection between the visual system and the muscular system, important for posture, is thought to involve the RF among other neural structures, highlighting the complex interplay between different parts of the nervous system in maintaining posture and balance [17].

Therefore, according to one theory, the connection between the visual organ and the muscular system occurs at the RF level [7]. Above all, this hypothesis concerns the middle part of the RF. It is responsible for processing sensory and motor information between the medulla oblongata and the higher parts of the brain. In this part, there are also centers that coordinate eye and facial movements, as well as processes related to sleep, such as rapid eye movement (REM) phases [16,17,18].

Closing and opening the eyes is associated with changes in ciliary muscle activity. Changes in the tension of the ciliary muscle cause a change in the shape of the eye’s lens [19]. The described muscle is an important part of the eye that controls aspects of accommodation [19,20]. The oculomotor nerve is responsible for its innervation [19]. The neural connections affecting motor control described above, and the anatomy of the RF as a site for combining signals from the visual organ and the muscular system, suggest the potential influence of accommodation as a factor influencing changes in the human muscular system.

The accommodation reflex is the visual response to focusing on near objects [20]. The accommodation reflex is made possible by changes in the tension of the ciliary muscles causing the lens of the eye to change shape [21]. The oculomotor nerve innervates the ciliary muscles [22]. Changes in the morphology of the ciliary muscle have been seen in people with myopia [23]. The accommodative response diminishes with age [24]. Generally, young adults have an accommodative response of 12–14 D; adults range between 4 and 8 D, and after the age of 50, the amplitude of accommodation declines to less than 2D [25]. Cycloplegia refers to the pharmacological paralysis of the ciliary muscles, and it results primarily in the inhibition of accommodation. Cyclopentolate is a muscarinic antagonist used as a cycloplegic and mydriatic eye drop [26]. After the instillation of cyclopentolate, cycloplegia typically lasts 6–24 h, while pupil dilation (mydriasis) typically lasts up to 24 h [27,28].

The aim of the present investigation was to pharmacologically induce accommodative paralysis and evaluate its effects on the bioelectrical activity of the muscular system. Firstly, it was decided to compare the bioelectrical activity before accommodation paralysis and then following unilateral and bilateral accommodation paralysis. Subsequently, based on electromyographic results, electromyographic patterns were computed utilizing current scientific literature. The hypothesis is that accommodation paralysis influences the bioelectric activity and electromyographic patterns of the muscular system. To standardize the results, electromyographic patterns were applied [29,30].

## 2. Materials and Methods

This study was conducted in accordance with the recommendations of the Declaration of Helsinki and was approved by the local Bioethics Committee (KE-0254/259/12/2022). All subjects were informed of the purpose of the study, the procedures, and the consequences of accommodative paralysis (including impaired vision, and they were recommended not to drive, operate machinery in motion, or work at height for 24 h after the drops were administered). All subjects were allowed to withdraw from this study at any time and gave written consent to participate in the study. The study protocol has been published [28].

This study included 16 people, who were divided into two groups. The first group with myopia included 8 subjects (4 women and 4 men, mean age 25.13 years) and the second emmetropic group also included 8 subjects (3 women and 5 men, mean age 24.75 years). The size of the groups was not statistically different (*p* = 0.28) and the groups were not statistically different in terms of age (*p* = 0.96) (Table 1).

Inclusion and exclusion criteria were applied according to the accepted study protocol [28]. Healthy subjects without temporomandibular disorders, refractive errors other than myopia, ocular disorders, malocclusion, current orthodontic treatment, and head and neck diseases were included in this study. In addition, an exclusion criterion was applied in the form of hypersensitivity to the active substance (cyclopentolate hydrochloride) and excipients: boric acid, potassium chloride, disodium edetate, sodium carbonate, benzalkonium chloride, sodium hydroxide 40%, hydrochloric acid 10%, sodium chloride. A complete list of the used exclusion and inclusion criteria can be found in the study protocol [28].

**Figure 1:**
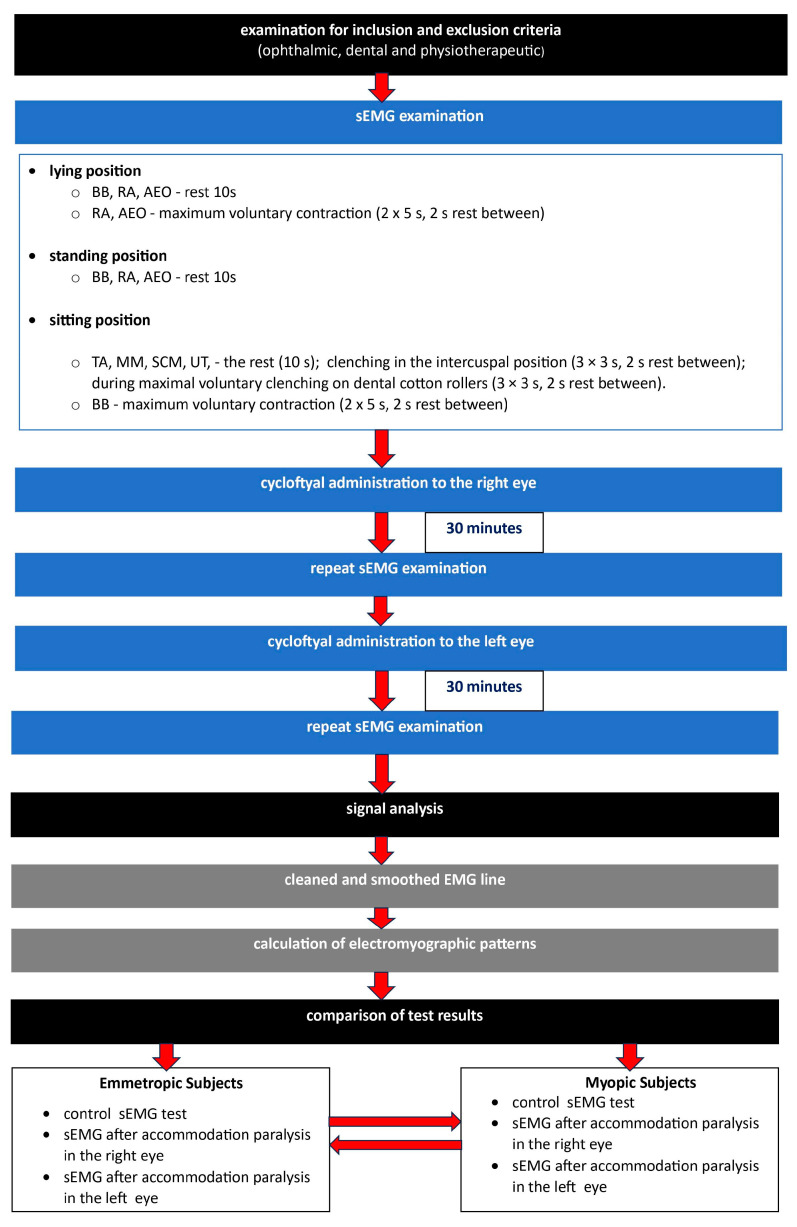
Diagram for conducting this study. TA—the anterior part of the temporalis muscle; MM—the superficial part of the masseter muscle; SCM—the middle part of the sternocleidomastoid muscle; UT—the upper part of the trapezius muscle; RA—the rectus abdominis muscle; BB—biceps brachii muscle; AEO—abdominal external oblique muscle.

This study was conducted as shown in Figure 1.

This study was conducted according to Figure 1. After analyzing the inclusion and exclusion criteria, qualified study participants underwent sEMG examinations.

**Figure 2:**
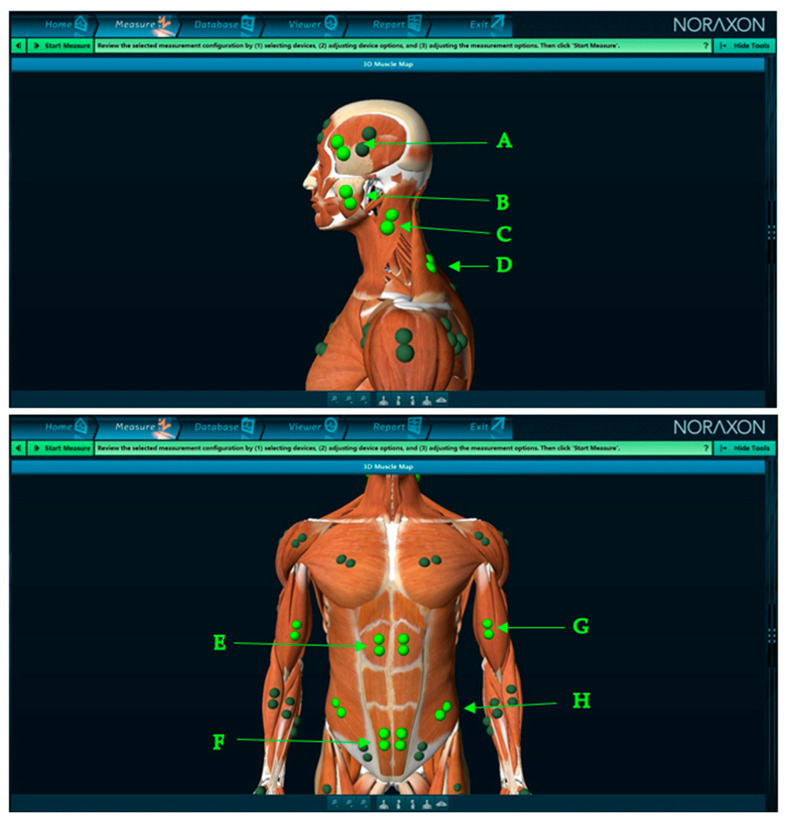
Electrode placement (screen from Noraxon MR3 3.18.08 software). A—the anterior part of the temporalis muscle (TA); B—the superficial part of the masseter muscle (MM); C—the middle part of the sternocleidomastoid muscle (SCM); D—the upper part of the trapezius muscle (UT); E—the upper part of the rectus abdominis muscle (RA-up); F—the lower part of the rectus abdominis muscle (RA-lo); G—biceps brachii muscle (BB); H—abdominal external oblique muscle (AEO).

The sEMG test was conducted using the Noraxon Ultium DTS 8-K MR 3 myo Muscle Master Edition (Scottsdale, AZ, USA). The following muscles were analyzed:

- The anterior part of the temporalis muscle (TA);
- The superficial part of the masseter muscle (MM);
- The middle part of the sternocleidomastoid muscle (SCM);
- The upper part of the trapezius muscle (UT);
- The upper part of the rectus abdominis muscle (RA-up);
- The lower part of the rectus abdominis muscle (RA-lo);
- Biceps brachii muscle (BB);
- Abdominal external oblique muscle (AEO) (Figure 2) [28].

The skin above the muscles under study was cleansed with 90% alcohol [30]. Electrodes (Ag/AgCl with a conductive surface of 16 mm) were placed according to the requirements of the SENIAM program (Figure 2). Patients had their eyes open during the examination. In the group with myopia, the sEMG study was conducted without refractive error correction during tests with opened eyes [30].

The tests were conducted in the lying, standing, and sitting positions. Recordings in the lying, standing, and sitting positions were obtained for the muscles BB, RA, and AEO. In each of these positions, a 10 s rest was recorded and during maximal voluntary contraction (2 × 5 s, with a 2 s rest between contractions) [28,30].

In the sitting position, additional muscles TA, MM, SCM, and UT were examined. Following the standard procedure for muscles of the masticatory system and cervical region, 3 procedures were performed: at the rest position of the mandible (10 s); during clenching in the intercuspal position (as hard as possible; 3 × 3 s, with a 2 s rest between contractions); during maximal voluntary clenching on dental cotton rollers (as hard as possible; 3 × 3 s, with a 2 s rest between contractions) [28,30].

After the sEMG tests, the application of a drug inducing accommodation paralysis was performed. Unilateral accommodation paralysis was initially induced (in the right eye). A full sEMG examination was repeated (Procedure 2), followed by accommodation paralysis in the left eye. Finally, the sEMG examination was repeated once more (Procedure 3).

**Figure 3:**
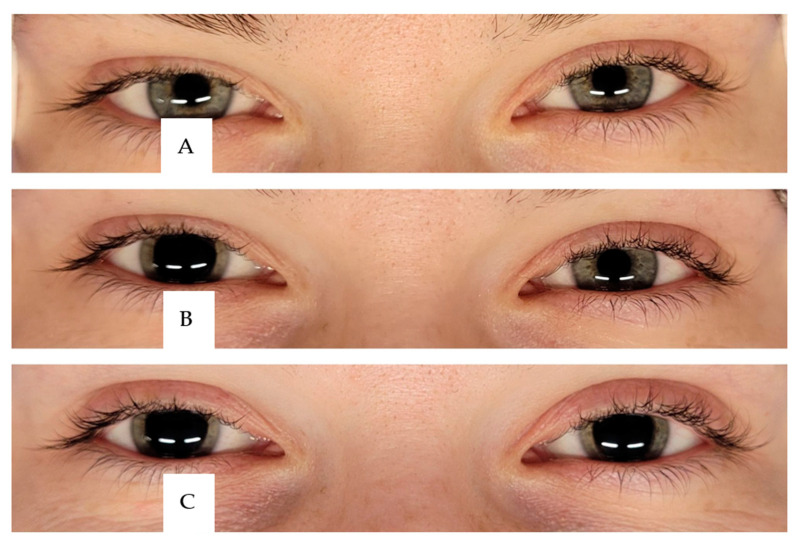
The course of mydriasis in this study. (**A**)—before the study; (**B**)—Cycloftyal is administered to the right eye and the sEMG test is repeated after 30 min. (**C**)—Cycloftyal is administered to the left eye and the sEMG is repeated after 30 min.

Cyclopentolate hydrochloride (Cycloftyal, 10 mg/mL, eye drops, solution, manufacturer Verco, Warsaw, Poland) was administered to paralyze accommodation initially to the right eye and then to the left eye. In our study, patients received one drop of the solution per eye. One drop of 1% Cycloftyal has a volume of about 0.03 mL, which means that one drop of the solution contains 0.3 mg of cyclopentolate hydrochloride [28]. One drop was instilled in the lower fornix of each participant’s anesthetized right and left eye, respectively. After instillation, punctal occlusion was conducted in order to prevent or minimize the amount of drug that could enter the systemic circulation (Figure 3) [31].

### 2.1. Data Analysis

Noraxon MR3 3.18.08 software was used to analyze the collected sEMG signals. This program was also used for signal processing. First, a researcher specializing in electromyography (first author) performed a visual analysis of the signal. The sample rate for sEMG was 2000 Hz, while for motion, it was 200 Hz. A high-pass filter cutoff value of 10 Hz and a low-pass filter cutoff of 500 Hz were applied to EMG signals. The analog output gain for EMG was set to 5 V. Subsequently, standard processing of the sEMG kinesiology signal in the form of line cleaning and smoothing was performed. This procedure was conducted with the dedicated MR3 3.18.08 software [30]. The obtained values were substituted into the following formulas.

From the obtained sEMG data, the following indices were calculated according to standard protocols for the masticatory and the cervical spine muscles [32,33,34,35,36]:

- AsI (asymmetry index) based on the following formula:

AsI = [(RMS_right_ − RMS_left_)/(RMS_right_ + RMS_left_)] × 100 (1)

- AcI (activity index) based on the following formula:

AcI = [(RMS_masseter_ − RMS_temporal_)/(RMS_masseter_ + RMS_temporal_)] × 100 (2)

- MVC (maximum voluntary contraction) based on the following formula:

MVC = [voluntary teeth clenching/voluntary teeth clenching on cotton rollers] × 100%(3)

- POC (percentage overlapping coefficient) based on the following formula:

POC = [(MM_right_ + TA_right_)/(MM_left_ + TA_left_)] × 100% (4)

- TC (torque coefficient) based on the following formula:

TC = [(TA_right_ + MM_left_) − (TA_left_ + MM_right_)] × 100% (5)

- Functional Clenching Index (FCI) based on the following formula:

FCI = Clenching muscle/REST_muscle_ (6)

- Functional Clenching Activity Index (FCAI) based on the following formula:

FCAI = [(FCI_MM-R or L_ − FCI _TA-R or L_)/(FCI _MM-R or L_ + FCI _TA-R or L_)] × 100 (7)

- Functional Clenching Symmetry Index (FCSI) based on the formula:

FCSI = [(FCI_muscle-R_ − FCI_muscle-L_)/(FCI_muscle-R_ + FCI_muscle-L_)] × 100 (8)

From the obtained sEMG data, the following indices were calculated according to standard protocols for postural muscles and arm muscles [35,36,37]:

- The percent of integrated EMG (iEMG) (%) of muscle participation in activity is based on the following formula:

Biceps brachii (BB) _right or left %_ = (RMS_right or left_ × 100%)/(RMS_right_ + RMS_left_) (9)

Rectus abdominis (RA) _right or left and up or low %_ = (RMS_right or left_ × 100%)/(RMS _up right_ + RMS _up left_ + RMS _low right_ + RMS _low left_) (10)

Abdominal external oblique (AEO) _right or left %_ = (RMS_right or left_ × 100%)/(RMS_right_ + RMS_left_) (11)

- FCI was adopted as a Functional Contraction Index (FCoI) based on the following formula:

FCoI = Contraction muscle/REST_muscle_ (12)

- FCSI was adopted as a Functional Contraction Symmetry Index (FCoSI) based on the following formula:

FCoSI = [(FCoI_muscle-R_ − FCoI_muscle-L_)/(FCoI_muscle-R_ + FCoI_muscle-L_)] × 100 (13)

AsI was counted according to the previously presented Formula (1).

### 2.2. Statistical Analysis

Statistical analysis was performed using Statistica software (version 13.3.721.1, StaSoft Poland TIBICO Software Inc., Palo Alto, CA, USA). The Chi-square test was used to compare the number of females and males in groups.

First, the normality of the distribution was verified using the Shapiro–Wilk test and the Kolmogorov–Smirnov test (with the Lilliefors correction). All distributions deviated from normal; therefore, it was decided to use non-parametric tests. The Mann–Whitney U test (Z) was used to analyze the two groups. Effect sizes were determined for *t*-tests using the Cohen d method and interpreted as small (0.2), medium (0.5), and large (0.8) effect sizes [38,39,40]. Statistical significance in this test was set at *p* ≤ 0.05. With this test, a Bonferroni correction (alpha = 0.05/3 = 0.0167) was introduced, based on which the significance level was set at *p* ≤ 0.0167.

Due to the number of performed analyses, the most important statistical results are presented below; a full description and complete analyses can be found in the Supplementary Materials.

## 3. Results

The groups were not statistically different in terms of the number of men and women, their age, and the range of mandibular mobility. There were differences only in the presence of a refractive error (Table 1).

### 3.1. Electromyographic Analysis of the Masticatory and Cervical Region

When comparing the activation of the masticatory and cervical spine muscles between Emmetropic Subjects and Myopic Subjects showed differences only in the bioelectrical activity of the SCM muscle (*p* = 0.04, ES = 0.48). After the paralysis of accommodation in the right eye, the differences between the groups were no longer significant; statistical significance returned during Procedure 3 (*p* = 0.01, ES = 0.58). During the last procedure, we observed the appearance of significant differences in MVC-SCM-R (*p* = 0.01, ES = 0.54) and MVC-SCM-L (*p* = 0.03, ES = 0.50) (Table 2).

No statistically significant changes were observed in rest and clenching on dental cotton rollers. Differences were observed in clenching in the intercuspal position. Statistically significant differences were observable in Procedure 2 in AcI-total (tot) (*p* = 0.03, ES = 0.50) and during the last procedure (no. 3), FCAI-R (*p* = 0.03, ES = 0.50), and FCI-SCM-L (*p* = 0.03, ES = 0.48) (Table 2). The values of the AsI range from +100% to −100%, where +100% indicates the involvement of the muscles exclusively on the right side during activity, and −100% indicates involvement exclusively on the left side. Meanwhile, a value of 0% for the asymmetry index denotes equal activity of the muscles on both the right and left sides [35].

It is worth noting that there were no statistically significant differences among Procedures 1–3 in the Emmetropic Subjects group and no statistically significant differences among Procedures 1–3 in the Myopic Subjects group (Supplementary Materials File S1).

### 3.2. Electromyographic Analysis of the Postural and Arm Muscles

Statistical analysis showed significant differences between groups in AEO-R (*p* = 0.01, ES = 0.60), AEO-R-% (*p* = 0.01, ES = 0.57), AEO-L-% (*p* = 0.01, ES = 0.57), and AsI-AEO (*p* = 0.01, ES = 0.57) in the lying position. Differences were not noticeable after the paralysis of accommodation in Procedure 2 and Procedure 3 (Table 3).

In the standing position, significant differences were noted in RA-Lo-R-% (*p* = 0.03, ES = 0.50) only in Procedure 3 (Table 3).

In the sitting position, significance appeared only during Procedure 2 (paralysis of right eye accommodation—Figure 2) in AEO-R-% (*p* = 0.04, ES = 0.47), AEO-L-% (*p* = 0.04, ES = 0.47), and AsI-AEO (*p* = 0.04, ES = 0.47) (Table 3). The values of the AcI range from +100% to −100%, where +100% indicates the exclusive involvement of the masseter muscle during activity, and −100% indicates the exclusive involvement of the temporal muscle [35].

During Procedure 1, statistically significant changes were observed in maximum voluntary contraction in BB-R (*p* = 0.03, ES = 0.50), FCI-BB-R (*p* = 0.02, *p* = 0.55), and FCI-RA-Lo-R (*p* = 0.04, ES = 0.47). Significances were not repeated in subsequent Procedures 2 and 3 (Table 3). Significant differences were observed in FCI-RA-Lo-L during Procedure 2 (*p* = 0.03 ES = 0.50) and Procedure 3 (*p* = 0.01, ES = 0.60). No differences were observed in FCI-RA-Lo-L during Procedure 1 (Table 3).

It is worth noting that there were no statistically significant differences among Procedures 1–3 in the Emmetropic Subjects group and no statistically significant differences among Procedures 1–3 in the Myopic Subjects group (Supplementary Materials File S1).

## 4. Discussion

The aim was to pharmacologically induce accommodative paralysis and evaluate its effects on the bioelectrical activity of the muscular system. The hypothesis is that accommodation paralysis influences the activity and electromyographic patterns of the muscular system. Based on the results obtained and the effect size of the differences obtained, we suggest confirming the null hypothesis. However, let us point out the need to examine the obtained results in a larger group.

The lack of statistically significant differences among Procedures 1–3 in the Emmetropic Subjects group and the lack of statistically significant differences among Procedures 1–3 in the Myopic Subjects group may show that compensation occurs. Rapid compensation on the part of the musculo-fascial system may be associated with the action of a small stimulus [41]. On the other hand, the observable differences between the Emmetropic and Myopic Subjects groups in Procedures 1–3 show different forms of muscle responses between the groups. It has been shown in earlier studies that the masticatory muscles react differently (to open and closed eyes) in Myopic Subjects compared to Emmetropic Subjects [7,8,42]. This may be related to the greater sensitivity of the nervous system of people with myopia. Earlier studies have shown that myopic people have higher central sensitization inventory scores compared to emmetropic people [43]. In addition, a case study showed that the correction of refractive error and the associated improvement in visual acuity (20/200, 20/40 20/20, 20/10) cause an increase in the bioelectrical activity of the masticatory muscles [44]. This confirms the possibility of a rapid response of the muscular system to changes in visual stimulus in people with myopia. The observed changes during Procedure 1 between groups (Emmetropic and Myopic Subjects) predominantly indicate changes in the musculoskeletal system caused by refractive error [7,8,42]. Changes were observed in the SCM, BB, AEO, and RA muscles. Changes in the SCM have already been seen in studies of people with myopia. Changes in the other muscle groups are likely to occur through the action of the musculo-fascial structures and their controlling nervous system [7,9].

During Procedure 2, the predominance of MM increased in AcI-tot subjects with myopia, and in Emmetropic Subjects, the results were closer to ideal symmetry. A similar situation occurred in AsI-AEO and FCI-RA-Lo-L. This confirms another strategy of muscular system advantage between groups. In Procedure 2 (paralysis of accommodation in the right eye), there was an increase in the percentage of AEO-R-% in both groups and a decrease in AEO-L-%. This suggests a potential effect of accommodation on bioelectrical activity.

In Procedure 3, in the masticatory and cervical spine muscles, the highest bioelectrical value and values of bioelectrical indices were observed in the Myopic Subjects group. In the Emmetropic Subjects group, a decrease in values was observed in all studied variables. It is worth noting that the postural and arm muscles showed the greatest decrease in bioelectrical values and values of bioelectrical indices in both groups.

The observable changes can be explained by the action of the nervous system and the associated fascial continuum [7]. The fascial continuum permeates and surrounds all muscles, organs and nerve fibers [45] and can also perform several other important functions including biomechanical force transmission, cellular signal transmission, and cell signaling [46]. The fascia is an abundantly innervated tissue; in particular, it contains proprioceptors and nociceptors [47]. Changes within one link of the fascial “chain” will affect the others [48]. Accommodation paralysis primarily affects the ciliary muscle of the eye [20,28]. The ciliary muscle connects to the choroid through the pars plana (orbicularis ciliaris) [49]. It then connects to the sclera, extraocular muscles, and Tenon’s capsule [50]. Tenon’s capsule surrounds the entire eyeball and extraocular muscles, starting from the optic nerve area [51]. It then connects to the levator palpebrae superioris muscle and then to the superficial musculoaponeurotic system (SMAS) [52]. Histological studies have shown that Tenon’s capsule is intimately connected to the outer episclera by delicate lamellae at the insertion of the extraocular muscles and to the dura mater around the optic nerve head [53]. The SMAS connects to platysma muscle [54]. Distally, the platysma muscle forms a continuity with the superficial fascia of the thorax and shoulder region [54,55]. The superficial thoracic fascia successively transitions to Scarpa’s fascia in the lower abdomen [54,56]. Scarpa’s fascia is a dense collagenous layer of connective tissue located in the anterior abdominal wall [56]. The description above shows the sequence of the fascia continuum. It is worth noting that muscle function cannot be separated from fascial function; there is a reciprocal effect on both entities [7,45,54]. The observed changes in muscle response between the groups (Procedures 2 and 3) can also be explained by changes in the first link in the chain described, i.e., the ciliary muscle. Wagner et al. found significant differences in ciliary muscle thickness, shape, and movement depending on the refractive error [23].

A paralysis of accommodation and, more specifically, a paralysis of the ciliary muscle will alter the reception of stimuli by the oculomotor nerve [22,57]. Different stimulus reception caused by accommodative paralysis can affect the RF. The RF contains functional groups of cells that are important for controlling eye and head movements [16]. In addition, there are hypotheses that the RF participates in accommodation [58].

In addition, it can be hypothesized that the RF responds to fascicular changes. It has been proven that the RF is involved in the transmission and modulation of nociceptive information [59]. Fascia has a rich innervation, and the number of nociceptors in it increases in pathological situations [47]. The RF is involved in extraocular muscle movements through gaze fixation and scapular movements [16,60]. According to a hypothesis developed by Simpkins, accommodation is a two-pronged process [61,62]. It involves changes in the lens and changes in the adducting and abducting external muscles. Muscle action stretches the elastin fibers behind the cornea [61,62]. It is worth noting that a very recent study (2023) found a negative correlation between inferior rectus muscle thickness and visual acuity, which may support the above description of the relationships. Furthermore, the same study found correlations between the extraocular muscles and the masticatory muscles, which may support the fascial pathway [9]. Again, here we can consider the combination of two systems in which a change in one part of the whole link changes the other. Whether it is a hypothetical change in stimuli perceived by the fascia receptors or a change in motor stimulus reception by the oculomotor nerve, changes occur throughout the human system.

The main practical aspect of this study is to demonstrate possible connections between the two systems. This will encourage interdisciplinary collaboration between specialists in the muscular system and specialists in eye diseases for the treatment of tension headaches in myopic individuals, for example.

The work has several limitations. First, it is a pilot study conducted on a small group. In the future, it should be conducted on a larger group. Because myopia is related to the human species, other species should be included in the future [63]. In addition, the reproducibility of the effect should be checked on other refractive errors. Among the strengths of this study is that it is the first of its kind in the world. In addition, statistically significant results have a medium effect size [64].

Based on the results and anatomical correlations, we suggest that accommodation influences the bioelectrical activity of the muscle system. It should be noted that, at present, it is not possible to conclude that the observed phenomenon is clinically relevant, and further research is recommended.

## Figures and Tables

**Table 1:** Presentation of groups.

		Emmetropic Subjects (*n* = 8)			Myopic Subjects (*n* = 8)				
		Mean		SD	Mean	SD	Test		*p*
	Female	4			3		x^2^		0.28
	Male	4			5				
	Age	25.13		1.55	24.75	2.19	Z	0.06	0.96
	Best Corrected Visual Acuity	R	n/a		1.0				n/a
		L	n/a		1.0				n/a
	Visual acuity	R	1.0		n/a				n/a
		L	1.0		n/a				n/a
	Refractive error (Dsph)	R	n/a		−3.25	1.79			n/a
		L	n/a		−2.78	0.86			n/a
	Intraocular Pressure (mmHg)	R	15.29	1.89	16.00	1.15	Z	−0.06	0.95
		L	16.14	2.04	15.25	1.26	Z	0.87	0.39
	Axial Length (mm)	R	24.71	0.67	23.86	0.40	Z	−1.31	0.19
		L	24.54	0.71	23.71	0.35	Z	−0.87	0.38
Mandibular Range of Motion (mm)	Pain-Free Opening	47.50		2.27	53.57	7.93	Z	−1.45	0.15
	Mandibular Movement to the Right	12.38		3.11	10.00	2.94	Z	1.51	0.13
	Mandibular Movement to the Left	11.88		2.85	10.43	2.94	Z	0.75	0.45
	Protrusion	9.67		2.25	7.83	1.94	Z	1.20	0.23

*n*—individuals in the sample; SD—standard deviation; R—right side; L—left side; Dsph—spherical diopter; mmHg—millimeters of mercury; μm—micrometer; mm—millimeter; x^2^—the Chi-square test; n/a—not applicable; Z—the Mann–Whitney U test.

**Table 2:** Presentation of statistically significant results of masticatory and cervical spine muscles.

		Emmetropic Subjects		Myopic Subjects		U	Z	*p*	ES
		Mean	SD	Mean	SD				
Maximum Voluntary Contraction									
Procedure 1	MVC-SCM-tot	65.07	31.85	145.51	119.07	17.00	−2.03	0.04 *	0.48
Procedure 2	MVC-SCM-tot	68.92	22.22	147.60	122.04	19.00	−1.85	0.06	
Procedure 3	MVC-SCM-tot	65.14	34.83	175.11	184.71	12.00	−2.47	0.01 *	0.58
Procedure 1	MVC-SCM-R	66.41	36.01	107.88	47.35	20.00	−1.77	0.08	
Procedure 2	MVC-SCM-R	67.72	22.09	174.99	228.12	18.00	−1.94	0.06	
Procedure 3	MVC-SCM-R	60.89	32.20	226.93	323.05	14.00	−2.30	0.02 *	0.54
Procedure 1	MVC-SCM-L	74.78	26.69	217.74	307.82	22.00	−1.59	0.11	
Procedure 2	MVC-SCM-L	72.34	28.23	122.75	77.23	28.00	−1.06	0.29	
Procedure 3	MVC-SCM-L	69.87	39.00	144.05	101.67	16.00	−2.12	0.03 *	0.50
Clenching in The Intercuspal Position									
Procedure 1	AcI-tot	11.41	15.30	27.63	19.49	19.00	−1.85	0.06	
Procedure 2	AcI-tot	−0.26	24.99	24.57	29.37	16.00	−2.12	0.03 *	0.50
Procedure 3	AcI-tot	−3.42	27.85	18.69	29.89	25.00	−1.32	0.19	
Procedure 1	FCAI-R	15.18	41.13	33.42	45.60	31.00	−0.79	0.43	
Procedure 2	FCAI-R	17.50	43.76	40.32	40.75	28.00	−1.06	0.29	
Procedure 3	FCAI-R	10.90	33.53	44.88	38.82	16.00	−2.12	0.03 *	0.50
Procedure 1	FCI-SCM-L	4.79	6.18	11.07	12.23	18.00	−1.94	0.06	
Procedure 2	FCI-SCM-L	5.12	6.86	7.44	5.20	23.00	−1.50	0.13	
Procedure 3	FCI-SCM-L	4.82	5.12	11.09	7.25	15.00	−2.21	0.03 *	0.48

*n*—individuals in the sample; SD—standard deviation; R—right side; L—left side; MVC—maximum voluntary contraction; SCM—the middle part of the sternocleidomastoid muscle; AcI—activity index; FCAI—Functional Clenching Activity Index; FCI—Functional Clenching Index; U—the difference between the two rank totals; Z—the Mann–Whitney U test; ES—effect size; * significant difference.

**Table 3:** Presentation of statistically significant results of the postural and arm muscles.

		Emmetropic Subjects		Myopic Subjects		U	Z	*p*	ES
		Mean	SD	Mean	SD				
Lying Position									
Procedure 1	AEO-R	6.71	5.16	3.46	2.30	7.00	−2.54	0.01 *	0.60
Procedure 2	AEO-R	7.26	11.07	3.33	1.30	26.00	−0.53	0.60	
Procedure 3	AEO-R	6.79	8.96	3.29	1.85	24.00	−0.74	0.46	
Procedure 1	AEO-R-%	55.24	11.06	36.17	10.05	8.00	−2.43	0.01 *	0.57
Procedure 2	AEO-R-%	53.06	15.34	42.87	14.53	24.00	−0.74	0.46	
Procedure 3	AEO-R-%	49.90	21.95	44.29	12.87	27.00	−0.42	0.67	
Procedure 1	AEO-L-%	44.76	11.06	56.82	16.33	8.00	2.43	0.01 *	0.57
Procedure 2	AEO-L-%	46.94	15.34	47.93	14.08	24.00	0.74	0.46	
Procedure 3	AEO-L-%	50.10	21.95	46.79	13.99	27.00	0.42	0.67	
Procedure 1	AsI-AEO	10.48	22.12	−15.16	29.01	8.00	−2.43	0.01 *	0.57
Procedure 2	AsI-AEO	6.12	30.68	−0.64	16.86	24.00	−0.74	0.46	
Procedure 3	AsI-AEO	−0.21	43.90	−0.45	11.53	27.00	−0.42	0.67	
Standing Position									
Procedure 1	RA-Lo-R-%	18.55	7.42	20.85	9.36	19.00	1.27	0.20	
Procedure 2	RA-Lo-R-%	18.38	5.66	20.85	7.96	19.00	1.27	0.20	
Procedure 3	RA-Lo-R-%	17.60	5.29	24.70	10.87	11.00	2.12	0.03 *	0.50
Sitting Position									
Procedure 1	AEO-R-%	46.86	6.42	35.16	14.22	19.00	−1.27	0.20	
Procedure 2	AEO-R-%	52.13	18.03	37.05	11.13	12.00	−2.01	0.04 *	0.47
Procedure 3	AEO-R-%	43.79	4.83	38.75	12.11	30.00	0.11	0.92	
Procedure 1	AEO-L-%	53.14	6.42	53.55	20.97	19.00	1.27	0.20	
Procedure 2	AEO-L-%	47.87	18.03	54.85	15.04	12.00	2.01	0.04 *	0.47
Procedure 3	AEO-L-%	56.21	4.83	52.02	15.66	30.00	−0.11	0.92	
Procedure 1	AsI-AEO	−6.28	12.83	−17.19	18.06	19.00	−1.27	0.20	
Procedure 2	AsI-AEO	4.27	36.06	−14.70	18.17	12.00	−2.01	0.04 *	0.47
Procedure 3	AsI-AEO	−12.42	9.66	−10.51	14.91	30.00	0.11	0.92	
Procedure 1	MVC-BB-R	477.53	359.67	825.12	471.41	11.00	2.12	0.03 *	0.50
Procedure 2	MVC-BB-R	558.33	351.20	683.55	341.43	23.00	0.85	0.40	
Procedure 3	MVC-BB-R	662.72	399.49	834.93	348.90	19.00	1.27	0.20	
Procedure 1	FCoI-BB-R	127.63	107.12	260.56	141.83	9.00	2.33	0.02 *	0.55
Procedure 2	FCoI-BB-R	157.82	126.51	218.48	155.73	20.00	1.16	0.24	
Procedure 3	FCoI-BB-R	181.54	148.94	305.95	153.26	14.00	1.80	0.07	
Procedure 1	FCoI-RA-lo-R	18.06	26.95	42.40	31.61	12.00	2.01	0.04 *	0.47
Procedure 2	FCoI-RA-lo-R	22.12	28.35	32.12	23.30	22.00	0.95	0.34	
Procedure 3	FCoI-RA-lo-R	24.38	39.19	39.51	29.38	14.00	1.80	0.07	
Procedure 1	FCoI-RA-lo-L	83.55	164.86	60.46	50.35	14.00	1.80	0.07	
Procedure 2	FCoI-RA-lo-L	35.50	84.69	47.09	33.40	11.00	2.12	0.03 *	0.50
Procedure 3	FCoI-RA-lo-L	43.88	102.44	56.61	40.90	7.00	2.54	0.01 *	0.60

*n*—individuals in the sample; SD—standard deviation; R—right side; L—left side; MVC—maximum voluntary contraction; AEO—abdominal external oblique muscle; AsI—asymmetry index; RA-Lo—the lower part of the rectus abdominis muscle; BB—biceps brachii muscle; FCoI— Functional Contraction Index; U—the difference between the two rank totals; Z—the Mann–Whitney U test; ES—effect size; * significant difference.

## Data Availability

For information on creating a dataset, contact the correspondence author or to the institutional body (Department of Sports Medicine, e-mail department.sports.medicine@umlub.pl).

## Supplementary Materials

The following supporting information can be downloaded at: https://www.mdpi.com/article/10.3390/diagnostics14090961/s1. File S1: Full results of the statistical analysis.

## Abbreviations

AcI	activity index
AEO	abdominal external oblique muscle
AsI	asymmetry index
BB	biceps brachii muscle
Dsph	spherical diopter
ES	effect size
FCAI	Functional Clenching Activity Index
FCI	Functional Clenching Index
FCoI	Functional Contraction Index
FCoSI	Functional Contraction Symmetry Index
FCSI	Functional Clenching Symmetry Index
iEMG	integrated EMG
L	left side
MM	the superficial part of the masseter muscle
mm	millimeter
mmHg	millimeters of mercury
MVC	maximum voluntary contraction
*n*	individuals in the sample
POC	percentage overlapping coefficient
R	right side
RA-lo	the lower part of the rectus abdominis muscle
RA-up	the upper part of the rectus abdominis muscle
RF	reticular formation
SCM	the middle part of the sternocleidomastoid muscle
SD	standard deviation
sEMG	surface electromyography
SMAS	the superficial musculoaponeurotic system
TA	the anterior part of the temporalis muscle
TC	torque coefficient
TTH	tension-type headaches
U	the difference between the two rank totals
UT	the upper part of the trapezius muscle
x^2^	the Chi-square test
Z	the Mann–Whitney U test
μm	micrometer
μV	microvolt
