# Supplementary material for: Pharmacologically Induced Accommodation Palsy and the Bioelectrical Activity of the Muscular System: A Preliminary Investigation
Source: Diagnostics (Basel). 2024 May 4;14(9):961. doi: 10.3390/diagnostics14090961 (PMC11082982; doi:10.3390/diagnostics14090961)
Supplement: Supplementary file 1 [file diagnostics-14-00961-s001.zip › diagnostics-2939891-supplementary.pdf]

Table S1. Presentation of groups. 2

Table S2. Comparison of bioelectrical activity findings of masticatory and cervical spine muscles in emmetropic subjects and myopic subjects (Without Paralysis of Accommodation). 3

Table S3. Comparison of bioelectrical activity findings of masticatory and cervical spine muscles in emmetropic subjects and myopic subjects (Accommodation Paralysis in The Right Eye). 6

Table S4. Comparison of bioelectrical activity findings of masticatory and cervical spine muscles in emmetropic subjects and myopic subjects (Accommodation Paralysis in The Left Eye). 9

Table S5. Comparison of bioelectrical activity findings of masticatory and cervical spine muscles in emmetropic subjects. 12

Table S6. Comparison of bioelectrical activity findings of masticatory and cervical spine muscles in myopic subjects. 15

Table S7. Comparison of bioelectrical activity findings of postural muscles and the arm muscles in emmetropic subjects and myopic subjects (Without Paralysis of Accommodation). 18

Table S8. Comparison of bioelectrical activity findings of postural muscles and the arm muscles in emmetropic subjects and myopic subjects (Accommodation Paralysis in The Right Eye). 21

Table S9. Comparison of bioelectrical activity findings of postural muscles and the arm muscles in emmetropic subjects and myopic subjects (Accommodation Paralysis in The Left Eye). 24

Table S10. Comparison of bioelectrical activity findings of postural muscles and the arm muscles in emmetropic subjects. 27

Table S11. Comparison of bioelectrical activity findings of postural muscles and the arm muscles in myopic subjects. 31

A

Table S1. Presentation of groups.

			Emmetropic Subjects (n=8)		Myopic Subjects (n=8)				
			**Mean**	**SD**	**Mean**	**SD**	**test**		**p**
	female		4		3		x^2		0.28
	male		4		5				
	age		25.13	1.55	24.75	2.19	Z	0.06	0.96
	Best Corrected Visual Acuity	R	n/a		1.0				n/a
		L	n/a		1.0				n/a
	Visual Acuity	R	1.0		n/a				n/a
		L	1.0		n/a				n/a
	Refractive Error (Dsph)	R	n/a		-3.25	1.79			n/a
		L	n/a		-2.78	0.86			n/a
	Intraocular Pressure (mmHg)	R	15.29	1.89	16.00	1.15	Z	-0.06	0.95
		L	16.14	2.04	15.25	1.26	Z	0.87	0.39
	Axial Length (mm)	R	24.71	0.67	23.86	0.40	Z	-1.31	0.19
		L	24.54	0.71	23.71	0.35	Z	-0.87	0.38
Mandibular Range Of Motion (mm)	Pain Free Opening		47.50	2.27	53.57	7.93	Z	-1.45	0.15
	Mandibular Movement to The Right		12.38	3.11	10.00	2.94	Z	1.51	0.13
	Mandibular Movement to The Left		11.88	2.85	10.43	2.94	Z	0.75	0.45
	Protrusion		9.67	2.25	7.83	1.94	Z	1.20	0.23

n—individuals in the sample; SD—standard deviation; R—right side; L—left side; Dsph—spherical diopter; mmHg—millimeters of mercury; μm—micrometer; mm—millimeter; x2—the Chi-square test; Z— the Mann-Whitney U test.

Table S2. Comparison of bioelectrical activity findings of masticatory and cervical spine muscles in emmetropic subjects and myopic subjects (Without Paralysis of Accommodation).

		**Emmetropic Subjects**		**Myopic Subjects**		**U**	**Z**	**P**
		Mean	SD	Mean	SD			
**Rest**	TA-R	5.39	2.74	6.49	5.38	39.00	-0.09	0.93
	TA-L	8.64	6.71	7.38	4.21	39.00	0.09	0.93
	TA-tot	7.02	3.99	6.93	4.54	39.00	0.09	0.93
	AsI-TA	-15.86	30.94	-13.02	31.95	37.00	-0.26	0.79
	MM-R	3.91	2.91	3.97	3.39	39.00	-0.09	0.93
	MM-L	3.77	3.83	5.86	8.66	37.00	-0.26	0.79
	MM-tot	3.84	3.29	4.91	5.95	38.00	-0.18	0.86
	AsI-MM	9.67	37.37	-7.00	22.67	31.00	0.79	0.43
	AcI-R	-18.14	38.49	-18.09	39.86	39.00	-0.09	0.93
	AcI-L	-36.77	41.07	-25.05	38.65	36.00	-0.35	0.72
	AcI-tot	-29.78	36.33	-23.96	34.16	33.00	-0.62	0.54
	POC	92.98	45.83	86.25	36.61	38.00	0.18	0.86
	TC	-337.67	634.41	99.56	620.33	29.50	-0.93	0.35
	SCM-R	3.66	1.41	2.89	1.61	20.00	1.77	0.08
	SCM-L	3.45	1.39	3.01	1.23	34.00	0.53	0.60
	SCM-tot	3.55	0.86	2.95	0.98	25.00	1.32	0.19
	AsI-SCM	3.52	26.76	-3.92	27.43	29.00	0.97	0.33
	UT-R	8.33	7.43	5.31	3.43	35.00	0.44	0.66
	UT-L	10.14	12.96	3.81	1.22	26.00	1.24	0.22
	UT-tot	9.24	9.45	4.56	1.70	22.00	1.59	0.11
	AsI-UT	-5.50	44.04	6.42	39.22	34.00	-0.53	0.60
**Clenching in The Intercuspal Position**	TA-R	178.86	81.31	142.63	43.19	30.00	0.88	0.38
	TA-L	167.54	88.51	150.33	52.85	38.00	0.18	0.86
	TA-tot	173.20	77.02	146.48	43.56	36.00	0.35	0.72
	AsI -TA	3.16	16.44	-2.73	15.28	36.00	0.35	0.72
	FCI-TA-R	40.12	24.78	41.58	31.35	40.00	0.00	1.00
	FCI-TA-L	30.80	28.34	30.53	23.97	40.00	0.00	1.00
	FCI-TA -tot	31.77	23.78	31.51	21.62	40.00	0.00	1.00
	FCSI-TA	17.16	35.12	10.38	34.45	37.00	0.26	0.79
	MM-R	220.49	162.32	297.11	204.74	31.00	-0.79	0.43
	MM-L	189.96	123.40	261.62	191.41	31.00	-0.79	0.43
	MM-tot	205.23	138.39	279.37	196.66	32.00	-0.71	0.48
	AsI -MM	2.78	17.87	3.89	14.50	40.00	0.00	1.00
	AcI-R	-1.63	29.26	22.78	34.00	21.00	-1.68	0.09
	AcI-L	-1.34	24.69	16.09	27.94	24.00	-1.41	0.16
	AcI- tot	-1.39	23.97	19.46	29.78	21.00	-1.68	0.09
	FCI-MM-R	89.03	91.11	128.46	120.79	33.00	-0.62	0.54
	FCI-MM-L	1869.68	5399.22	90.51	89.03	39.00	0.09	0.93
	FCI-MM-tot	81.57	69.38	104.47	100.56	37.00	-0.26	0.79
	FCSI-MM	-9.69	43.48	10.49	31.84	28.00	-1.06	0.29
	FCAI-R	15.18	41.13	33.42	45.60	31.00	-0.79	0.43

	FCAI-L	31.13	51.14	39.47	33.91	40.00	0.00	1.00
	FCAI-tot	25.93	43.97	38.81	35.48	32.00	-0.71	0.48
	POC	109.84	22.45	106.75	22.74	31.00	0.79	0.43
	TC	-1921.48	11549.16	-4318.59	6235.52	39.00	0.09	0.93
	SCM-R	11.73	7.92	18.98	12.58	27.00	-1.15	0.25
	SCM-L	12.61	12.66	27.54	24.85	18.00	-1.94	0.05
	SCM-tot	12.17	10.00	23.26	16.55	20.00	-1.77	0.08
	ASI-SCM	1.25	15.73	-13.89	22.08	24.00	1.41	0.16
	FCI-SCM-R	3.46	2.38	8.32	7.47	20.00	-1.77	0.08
	FCI-SCM-L	4.79	6.18	11.07	12.23	18.00	-1.94	0.06
	FCI-SCM- tot	3.93	3.94	8.44	6.20	20.00	-1.77	0.08
	FCSI-SCM	-2.39	27.06	-7.45	33.26	39.00	-0.09	0.93
	UT-R	7.67	5.95	6.32	5.04	34.00	0.53	0.60
	UT-L	9.97	9.39	4.60	1.23	23.00	1.50	0.13
	UT-tot	8.82	6.32	5.46	2.54	29.00	0.97	0.33
	ASI-UT	-12.79	32.67	2.05	41.52	37.00	-0.26	0.79
	FCI-UT-R	1.29	1.14	1.16	0.38	33.00	-0.62	0.54
	FCI-UT-L	1.51	1.22	1.31	0.56	35.00	-0.05	0.96
	FCI-UT-tot	1.10	0.34	1.23	0.42	39.00	-0.09	0.93
	FCSI-UT	7.88	48.49	-5.25	16.88	31.00	0.79	0.43
**Clenching on Dental Cotton Rollers**	TA-R	179.70	46.86	140.31	56.45	22.00	1.59	0.11
	TA-L	171.31	65.77	140.89	44.62	28.00	1.06	0.29
	TA-tot	175.51	46.69	140.60	36.67	23.00	1.50	0.13
	AsI -TA	4.14	19.12	-1.82	23.31	33.00	0.62	0.54
	FCI-TA-R	39.60	18.38	40.29	32.37	36.00	0.35	0.72
	FCI-TA-L	32.24	23.91	25.99	17.14	35.00	0.44	0.66
	FCI-TA -tot	33.06	19.44	29.04	19.04	37.00	0.26	0.79
	FCSI-TA	18.46	31.00	10.76	35.17	34.00	0.53	0.60
	MM-R	243.19	119.93	273.97	116.03	28.00	-1.06	0.29
	MM-L	218.81	78.91	258.29	109.04	31.00	-0.79	0.43
	MM-tot	231.00	96.87	266.13	104.66	30.00	-0.88	0.38
	AsI -MM	2.81	14.60	0.71	19.03	34.00	0.53	0.60
	AcI-R	10.92	22.73	28.42	28.68	23.00	-1.50	0.13
	AcI-L	12.50	17.86	26.90	17.66	27.00	-1.15	0.25
	AcI- tot	11.41	15.30	27.63	19.49	19.00	-1.85	0.06
	FCI-MM-R	88.98	73.72	107.09	74.80	34.00	-0.53	0.60
	FCI-MM-L	3291.50	9653.32	85.99	50.31	40.00	0.00	1.00
	FCI-MM-tot	90.11	68.10	92.54	59.53	37.00	-0.26	0.79
	FCSI-MM	-6.97	40.75	7.46	33.44	33.00	-0.62	0.54
	FCAI-R	26.93	35.05	40.69	37.12	30.00	-0.88	0.38
	FCAI-L	42.12	46.69	47.49	37.30	39.00	-0.09	0.93
	FCAI-tot	37.60	38.42	46.81	30.18	37.00	-0.26	0.79
	POC	110.09	23.08	106.72	36.07	31.00	0.79	0.43
	TC	-1597.41	9833.20	-1625.56	8668.20	37.00	-0.26	0.79
	SCM-R	32.64	51.80	17.98	10.95	38.00	0.18	0.86
	SCM-L	15.47	9.79	18.26	13.04	34.00	-0.53	0.60

SCM-tot	24.05	26.47	18.12	11.54	38.00	0.18	0.86
AsI -SCM	11.56	33.94	-0.44	19.87	33.00	0.62	0.54
FCI-SCM-R	9.50	14.41	8.44	8.76	40.00	0.00	1.00
FCI-SCM-L	5.45	4.93	6.66	5.30	33.00	-0.62	0.54
FCI-SCM- tot	7.39	8.12	7.14	6.49	38.00	-0.18	0.86
FCSI-SCM	8.08	41.04	2.83	25.05	39.00	-0.09	0.93
UT-R	10.27	7.09	10.76	6.72	36.00	-0.35	0.72
UT-L	7.25	2.91	7.18	4.42	34.00	0.53	0.60
UT-tot	8.76	4.38	8.97	4.85	37.00	0.26	0.79
AsI -UT	10.48	25.49	15.08	38.46	36.00	-0.35	0.72
FCI-UT-R	2.06	1.93	2.77	2.70	34.00	-0.53	0.60
FCI-UT-L	1.44	1.11	1.78	0.78	28.00	-1.06	0.29
FCI-UT-tot	1.25	0.66	1.88	1.21	26.00	-1.24	0.22
FCSI-UT	15.30	40.19	8.38	28.21	32.00	0.71	0.48
**MVC-TA-R**	99.03	38.02	110.88	43.36	35.00	-0.44	0.66
**MVC-TA-L**	98.33	32.12	113.01	49.11	30.00	-0.88	0.38
**MVC-TA-tot**	97.81	34.47	109.56	44.45	31.00	-0.79	0.43
**MVC-MM-R**	87.21	52.65	103.40	61.85	37.00	-0.26	0.79
**MVC-MM-L**	82.97	48.53	97.78	57.83	32.00	-0.71	0.48
**MVC-MM-tot**	84.76	49.10	99.95	59.68	33.00	-0.62	0.54
**MVC-SCM-R**	66.41	36.01	107.88	47.35	20.00	-1.77	0.08
**MVC-SCM-L**	74.78	26.69	217.74	307.82	22.00	-1.59	0.11
**MVC-SCM-tot**	65.07	31.85	145.51	119.07	17.00	-2.03	0.04*
**MVC-UT-R**	77.76	32.93	70.34	47.94	32.00	0.71	0.48
**MVC-UT-L**	151.93	168.84	90.29	66.86	27.00	1.15	0.25
**MVC-UT-tot**	107.84	80.67	77.83	57.72	26.00	1.24	0.22

AcI— activity index; AEO— abdominal external oblique muscle; AsI— asymmetry index; BB — biceps brachii muscle; ES—effect size; FCAI— Functional Clenching Activity Index; FCI — Functional Clenching Index; FCoI— Functional Contraction Index; FCoSI— Functional Contraction Symmetry Index; FCSI— Functional Clenching Symmetry Index; L— left side; MM — the superficial part of the masseter muscle; MVC— maximum voluntary contraction; n— individuals in the sample; POC— percentage overlapping coefficient; R— right side; RA-lo — the lower part of the rectus abdominis muscle; RA-up— the upper part of the rectus abdominis muscle; SCM — the middle part of the sternocleidomastoid muscle; SD — standard deviation; TA — the anterior part of the temporalis muscle; TC— torque coefficient; U— the difference between the two rank totals; UT— the upper part of the trapezius muscle; Z— the Mann-Whitney U test; * significant difference.

Table S3. Comparison of bioelectrical activity findings of masticatory and cervical spine muscles in emmetropic subjects and myopic subjects (Accommodation Paralysis in The Right Eye).

		**Emmetropic Subjects**		**Myopic Subjects**		**U**	**Z**	**P**
		Mean	SD	Mean	SD			
**Rest**	TA-R	4.94	1.79	7.31	5.91	37.00	-0.26	0.79
	TA-L	7.10	3.48	7.02	4.50	36.00	0.35	0.72
	TA-tot	6.02	1.73	7.17	5.06	37.00	0.26	0.79
	AsI -TA	-14.30	28.76	-5.23	24.10	34.00	-0.53	0.60
	MM-R	3.91	3.49	3.37	1.63	40.00	0.00	1.00
	MM-L	4.73	4.01	6.31	9.96	34.00	0.53	0.60
	MM-tot	4.32	3.73	4.84	5.52	37.00	-0.26	0.79
	AsI -MM	-10.41	13.24	-9.00	33.79	36.00	-0.35	0.72
	AcI-R	-18.12	33.52	-24.37	35.68	34.00	0.53	0.60
	AcI-L	-22.12	33.63	-20.27	39.08	40.00	0.00	1.00
	AcI-tot	-22.52	31.79	-20.82	30.51	36.00	-0.35	0.72
	POC	82.55	33.96	88.89	27.59	34.00	-0.53	0.60
	TC	-134.22	449.11	323.44	1133.55	32.00	-0.71	0.48
	SCM-R	3.47	1.52	4.24	3.81	40.00	0.00	1.00
	SCM-L	4.22	3.37	3.05	1.04	37.00	0.26	0.79
	SCM-tot	3.84	1.93	3.64	1.95	38.00	0.18	0.86
	AsI -SCM	-3.86	25.84	6.70	26.14	37.00	-0.26	0.79
	UT-R	5.39	5.17	6.64	6.64	38.00	-0.18	0.86
	UT-L	5.40	3.47	4.88	3.07	40.00	0.00	1.00
	UT-tot	5.39	3.85	5.76	3.77	38.00	-0.18	0.86
	AsI -UT	-6.07	32.02	1.46	43.23	37.00	-0.26	0.79
**Clenching in The Intercuspal Position**	TA-R	168.65	79.71	145.50	45.88	34.00	0.53	0.60
	TA-L	161.52	86.57	130.69	69.60	37.00	0.26	0.79
	TA-tot	165.09	75.66	138.09	44.35	30.00	0.88	0.38
	AsI -TA	1.72	18.56	8.73	36.70	38.00	-0.18	0.86
	FCI-TA-R	37.08	19.83	38.88	30.06	38.00	0.18	0.86
	FCI-TA-L	29.22	29.05	26.68	21.59	38.00	0.18	0.86
	FCI-TA -tot	30.39	22.62	30.23	20.49	40.00	0.00	1.00
	FCSI-TA	15.03	31.78	12.57	41.76	35.00	0.44	0.66
	MM-R	225.16	179.85	302.33	204.12	32.00	-0.71	0.48
	MM-L	186.74	124.11	284.64	208.67	31.00	-0.79	0.43
	MM-tot	205.95	148.79	293.48	205.02	32.00	-0.71	0.48
	AsI -MM	4.30	15.90	2.65	11.26	37.00	0.26	0.79
	AcI-R	0.94	29.39	23.55	31.70	24.00	-1.41	0.16
	AcI-L	-1.19	26.52	29.34	37.79	18.00	-1.94	0.05
	AcI- tot	-0.26	24.99	24.57	29.37	16.00	-2.12	0.03*
	FCI-MM-R	89.59	88.90	126.61	122.52	32.00	-0.71	0.48
	FCI-MM-L	55.94	41.27	87.44	79.95	31.00	-0.79	0.43
	FCI-MM-tot	67.52	53.07	97.55	88.19	33.00	-0.62	0.54
	FCSI-MM	14.14	24.72	11.84	35.92	40.00	0.00	1.00
	FCAI-R	17.50	43.76	40.32	40.75	28.00	-1.06	0.29
	FCAI-L	18.58	46.78	45.49	34.74	27.00	-1.15	0.25

	FCAI-tot	20.62	43.40	41.74	28.82	30.00	-0.88	0.38
	POC	109.61	23.44	116.49	44.67	31.00	0.79	0.43
	TC	-3128.33	11170.30	-288.00	8012.85	28.00	-1.06	0.29
	SCM-R	13.03	12.47	37.80	57.72	21.00	-1.68	0.09
	SCM-L	16.09	18.54	19.96	9.49	23.50	-1.46	0.15
	SCM-tot	14.56	15.37	28.88	29.41	19.00	-1.85	0.06
	AsI -SCM	-5.36	19.20	6.14	34.81	27.00	-1.15	0.25
	FCI-SCM-R	4.00	4.13	14.14	23.72	26.00	-1.24	0.22
	FCI-SCM-L	5.12	6.86	7.44	5.20	23.00	-1.50	0.13
	FCI-SCM- tot	4.50	5.46	10.14	11.58	21.00	-1.68	0.09
	FCSI-SCM	-0.76	19.04	-1.84	42.26	39.00	0.09	0.93
	UT-R	10.28	9.54	8.72	7.70	34.00	0.53	0.60
	UT-L	6.35	3.73	6.75	3.39	40.00	0.00	1.00
	UT-tot	8.31	6.05	7.73	5.23	39.00	0.09	0.93
	ASI-UT	8.65	27.71	-2.53	33.79	30.00	0.88	0.38
	FCI-UT-R	2.54	2.71	1.34	0.38	39.00	-0.09	0.93
	FCI-UT-L	1.30	0.64	1.57	0.91	24.00	-1.41	0.16
	FCI-UT-tot	1.73	1.49	1.34	0.39	40.00	0.00	1.00
	FCSI-UT	15.65	25.52	-3.89	22.41	22.00	1.59	0.11
**Clenching on Dental Cotton Rollers**	TA-R	190.23	70.03	162.56	65.42	31.00	0.79	0.43
	TA-L	179.31	91.68	153.40	43.72	34.00	0.53	0.60
	TA-tot	184.77	75.82	157.98	44.34	31.00	0.79	0.43
	AsI -TA	5.76	17.90	0.64	19.91	36.00	0.35	0.72
	FCI-TA-R	41.80	17.23	43.93	39.66	34.00	0.53	0.60
	FCI-TA-L	33.50	29.23	30.98	19.97	39.00	-0.09	0.93
	FCI-TA -tot	34.94	22.02	35.04	26.24	37.00	0.26	0.79
	FCSI-TA	19.08	30.49	5.79	27.10	28.00	1.06	0.29
	MM-R	290.44	185.96	307.34	140.65	35.00	-0.44	0.66
	MM-L	257.19	138.20	290.20	147.52	35.00	-0.44	0.66
	MM-tot	273.81	157.72	298.77	138.10	36.00	-0.35	0.72
	AsI -MM	3.86	17.75	1.60	18.06	35.00	0.44	0.66
	AcI-R	14.28	25.11	26.64	26.74	29.00	-0.97	0.33
	AcI-L	16.40	21.49	26.24	17.83	31.00	-0.79	0.43
	AcI- tot	15.39	19.82	26.44	20.38	26.00	-1.24	0.22
	FCI-MM-R	109.62	89.82	114.68	76.12	37.00	-0.26	0.79
	FCI-MM-L	71.59	40.86	92.48	60.61	32.00	-0.71	0.48
	FCI-MM-tot	85.54	55.99	93.49	53.29	38.00	-0.18	0.86
	FCSI-MM	13.89	24.92	9.69	41.43	35.00	0.44	0.66
	FCAI-R	28.44	39.23	44.23	34.20	29.00	-0.97	0.33
	FCAI-L	32.79	37.97	43.61	34.87	34.00	-0.53	0.60
	FCAI-tot	33.53	34.89	44.00	25.73	36.00	-0.35	0.72
	POC	113.99	28.40	109.78	39.49	32.00	0.71	0.48
	TC	-2233.70	11314.51	-797.78	6085.07	34.00	-0.53	0.60
	SCM-R	17.52	11.15	19.18	7.45	34.00	-0.53	0.60
	SCM-L	21.47	20.50	19.30	11.26	36.00	-0.35	0.72
	SCM-tot	19.49	15.13	19.24	8.70	36.00	-0.35	0.72
	AsI -SCM	-3.44	20.47	2.26	20.94	38.00	-0.18	0.86
	FCI-SCM-R	5.21	3.31	6.23	3.77	35.00	-0.44	0.66

FCI-SCM-L	6.84	7.60	6.59	3.18	32.00	-0.71	0.48
FCI-SCM- tot	5.92	5.46	6.13	3.30	35.00	-0.44	0.66
FCSI-SCM	0.10	26.70	-5.20	32.09	39.00	0.09	0.93
UT-R	9.97	8.36	11.09	7.70	33.00	-0.62	0.54
UT-L	7.41	4.18	7.79	5.40	40.00	0.00	1.00
UT-tot	8.69	5.66	9.44	5.77	38.00	-0.18	0.86
AsI-UT	3.36	24.10	11.20	37.39	36.00	-0.35	0.72
FCI-UT-R	2.75	3.15	2.67	2.62	40.00	0.00	1.00
FCI-UT-L	1.51	0.64	1.73	1.13	39.00	0.09	0.93
FCI-UT-tot	1.14	0.34	1.45	0.80	33.00	-0.62	0.54
FCSI-UT	9.88	28.59	5.72	42.58	29.00	0.97	0.33
**MVC-TA-R**	89.93	38.46	98.53	42.11	36.00	-0.35	0.72
**MVC-TA-L**	94.72	33.43	92.29	53.20	38.00	0.18	0.86
**MVC-TA-tot**	91.91	35.62	94.08	44.38	38.00	0.18	0.86
**MVC-MM-R**	72.15	37.63	95.90	54.80	33.00	-0.62	0.54
**MVC-MM-L**	70.94	34.79	97.02	57.15	25.00	-1.32	0.19
**MVC-MM-tot**	71.02	34.99	95.27	55.20	30.00	-0.88	0.38
**MVC-SCM-R**	67.72	22.09	174.99	228.12	18.00	-1.94	0.06
**MVC-SCM-L**	72.34	28.23	122.75	77.23	28.00	-1.06	0.29
**MVC-SCM-tot**	68.92	22.22	147.60	122.04	19.00	-1.85	0.06
**MVC-UT-R**	99.89	33.11	92.62	65.06	33.00	0.62	0.54
**MVC-UT-L**	87.12	20.03	116.61	85.15	34.00	-0.53	0.60
**MVC-UT-tot**	94.00	23.70	95.15	58.48	33.00	0.62	0.54

AcI— activity index; AEO— abdominal external oblique muscle; AsI— asymmetry index; BB — biceps brachii muscle; ES—effect size; FCAI— Functional Clenching Activity Index; FCI — Functional Clenching Index; FCoI— Functional Contraction Index; FCoSI— Functional Contraction Symmetry Index; FCSI— Functional Clenching Symmetry Index; L— left side; MM — the superficial part of the masseter muscle; MVC— maximum voluntary contraction; n— individuals in the sample; POC— percentage overlapping coefficient; R— right side; RA-lo — the lower part of the rectus abdominis muscle; RA-up— the upper part of the rectus abdominis muscle; SCM — the middle part of the sternocleidomastoid muscle; SD — standard deviation; TA — the anterior part of the temporalis muscle; TC— torque coefficient; U— the difference between the two rank totals; UT— the upper part of the trapezius muscle; Z— the Mann-Whitney U test; * significant difference.

Table S4. Comparison of bioelectrical activity findings of masticatory and cervical spine muscles in emmetropic subjects and myopic subjects (Accommodation Paralysis in The Left Eye).

		**Emmetropic Subjects**		**Myopic Subjects**		**U**	**Z**	**P**
		Mean	SD	Mean	SD			
**Rest**	TA-R	4.99	2.46	7.03	5.35	35.50	-0.40	0.69
	TA-L	6.47	3.47	7.11	3.60	34.00	-0.53	0.60
	TA-tot	5.73	2.24	7.07	4.16	36.00	-0.35	0.72
	AsI -TA	-9.34	30.08	-7.22	25.10	40.00	0.00	1.00
	MM-R	4.00	2.49	3.21	2.05	34.50	0.49	0.63
	MM-L	3.99	2.45	6.39	11.12	31.00	0.79	0.43
	MM-tot	4.00	2.28	4.80	6.44	28.00	1.06	0.29
	AsI -MM	-1.86	24.63	-10.60	27.35	34.00	0.53	0.60
	AcI-R	-13.11	28.54	-29.20	35.68	27.00	1.15	0.25
	AcI-L	-20.94	32.47	-26.99	39.13	35.00	0.44	0.66
	AcI-tot	-18.65	25.53	-27.00	35.37	31.00	0.79	0.43
	POC	94.08	37.71	89.24	38.89	36.00	0.35	0.72
	TC	-149.89	433.21	310.22	1198.30	32.00	-0.71	0.48
	SCM-R	4.00	1.75	4.45	6.60	20.00	1.77	0.08
	SCM-L	3.29	0.80	2.66	0.63	20.00	1.77	0.08
	SCM-tot	3.64	1.18	3.56	3.27	18.00	1.94	0.05
	AsI -SCM	6.82	16.34	1.65	33.94	29.00	0.97	0.33
	UT-R	6.80	5.99	8.36	6.94	32.00	-0.71	0.48
	UT-L	28.01	64.22	5.25	2.94	30.00	0.88	0.38
	UT-tot	17.40	31.35	6.81	4.63	34.00	0.53	0.60
	AsI -UT	-13.03	39.65	10.71	36.98	28.00	-1.06	0.29
**Clenching in The Intercuspal Position**	TA-R	188.30	82.96	155.70	53.57	33.00	0.62	0.54
	TA-L	186.69	88.70	158.86	59.84	37.50	0.22	0.83
	TA-tot	187.49	76.59	157.28	47.54	32.00	0.71	0.48
	AsI -TA	1.08	19.78	-1.25	18.92	39.00	0.09	0.93
	FCI-TA-R	50.20	36.00	36.78	23.77	33.00	0.62	0.54
	FCI-TA-L	46.40	47.36	29.89	21.32	36.00	0.35	0.72
	FCI-TA -tot	44.35	39.29	31.77	20.78	35.00	0.44	0.66
	FCSI-TA	9.77	36.01	5.63	27.37	39.00	0.09	0.93
	MM-R	236.27	187.08	326.11	239.26	30.00	-0.88	0.38
	MM-L	199.55	130.05	276.94	221.89	33.00	-0.62	0.54
	MM-tot	217.91	155.30	301.53	228.79	33.00	-0.62	0.54
	AsI -MM	3.69	15.61	7.92	13.71	33.00	-0.62	0.54
	AcI-R	-2.13	31.27	23.28	32.19	22.00	-1.59	0.11
	AcI-L	-4.04	31.03	14.09	30.48	27.00	-1.15	0.25
	AcI- tot	-3.42	27.85	18.69	29.89	25.00	-1.32	0.19
	FCI-MM-R	92.22	94.61	146.85	144.60	32.00	-0.71	0.48
	FCI-MM-L	59.47	43.28	109.72	119.72	32.00	-0.71	0.48
	FCI-MM-tot	70.85	60.97	123.98	132.11	33.00	-0.62	0.54
	FCSI-MM	5.57	33.02	16.91	33.05	34.00	-0.53	0.60
	FCAI-R	10.90	33.53	44.88	38.82	16.00	-2.12	0.03*
	FCAI-L	14.21	38.64	37.77	38.68	26.00	-1.24	0.22

	FCAI-tot	13.94	30.84	40.87	34.05	21.00	-1.68	0.09
	POC	106.91	21.51	112.58	28.64	38.00	0.18	0.86
	TC	-3510.74	12302.01	-5234.44	7683.73	39.00	-0.09	0.93
	SCM-R	14.78	12.90	59.02	107.21	22.00	-1.59	0.11
	SCM-L	16.70	19.16	28.06	17.40	21.00	-1.68	0.09
	SCM-tot	15.74	15.73	43.54	58.20	19.00	-1.85	0.06
	AsI -SCM	-1.02	17.53	3.61	33.82	39.00	0.09	0.93
	FCI-SCM-R	3.87	3.66	21.53	35.31	18.00	-1.94	0.05
	FCI-SCM-L	4.82	5.12	11.09	7.25	15.00	-2.21	0.03*
	FCI-SCM- tot	4.32	4.43	16.26	21.16	19.00	-1.85	0.06
	FCSI-SCM	-7.72	13.78	1.40	41.22	32.00	-0.71	0.48
	UT-R	8.58	7.08	7.84	5.04	40.00	0.00	1.00
	UT-L	7.26	4.23	6.01	2.73	34.00	0.53	0.60
	UT-tot	7.92	5.14	6.93	2.65	38.00	0.18	0.86
	AsI -UT	1.79	32.24	4.23	41.35	40.00	0.00	1.00
	FCI-UT-R	1.42	0.54	1.09	0.36	30.00	0.88	0.38
	FCI-UT-L	1.19	0.69	1.29	0.47	36.00	-0.35	0.72
	FCI-UT-tot	1.17	0.53	1.18	0.36	39.00	0.09	0.93
	FCSI-UT	10.39	38.79	-7.20	16.80	26.00	1.24	0.22
**Clenching on Dental Cotton Rollers**	TA-R	206.81	62.67	171.46	65.71	30.00	0.88	0.38
	TA-L	190.63	72.09	160.15	47.38	33.00	0.62	0.54
	TA-tot	198.72	59.45	165.80	41.62	33.00	0.62	0.54
	AsI -TA	5.34	17.42	1.43	21.76	35.00	0.44	0.66
	FCI-TA-R	51.39	29.41	41.01	32.14	31.00	0.79	0.43
	FCI-TA-L	47.72	46.43	28.44	15.65	35.00	0.44	0.66
	FCI-TA -tot	46.62	37.29	32.41	19.81	33.00	0.62	0.54
	FCSI-TA	13.90	29.69	7.73	29.97	40.00	0.00	1.00
	MM-R	304.64	159.72	309.06	150.30	39.00	-0.09	0.93
	MM-L	258.93	117.29	287.96	148.07	37.00	-0.26	0.79
	MM-tot	281.79	133.57	298.51	143.68	37.00	-0.26	0.79
	AsI -MM	5.65	17.31	1.93	18.00	32.00	0.71	0.48
	AcI-R	13.77	25.27	23.74	27.73	33.00	-0.62	0.54
	AcI-L	13.67	22.56	23.34	22.32	30.00	-0.88	0.38
	AcI- tot	13.82	20.21	23.41	22.72	29.00	-0.97	0.33
	FCI-MM-R	113.96	95.55	127.23	98.96	36.00	-0.35	0.72
	FCI-MM-L	76.54	32.95	108.20	87.51	32.00	-0.71	0.48
	FCI-MM-tot	87.93	56.05	112.28	91.00	36.00	-0.35	0.72
	FCSI-MM	7.95	36.07	10.94	35.63	35.00	0.44	0.66
	FCAI-R	25.75	28.25	46.63	34.66	25.00	-1.32	0.19
	FCAI-L	30.30	37.02	45.41	39.42	30.00	-0.88	0.38
	FCAI-tot	30.32	24.32	45.93	29.67	22.00	-1.59	0.11
	POC	115.22	25.76	110.21	39.53	31.00	0.79	0.43
	TC	-2953.70	10727.11	-978.52	8422.87	35.00	-0.44	0.66
	SCM-R	23.18	14.18	23.51	16.70	37.00	0.26	0.79
	SCM-L	23.19	21.48	20.88	8.23	35.00	-0.44	0.66
	SCM-tot	23.18	17.51	22.20	9.39	34.00	-0.53	0.60
	AsI-SCM	4.65	14.05	-0.39	29.02	26.00	1.24	0.22
	FCI-SCM-R	6.76	5.31	10.56	9.25	32.00	-0.71	0.48

FCI-SCM-L	7.03	5.88	7.73	2.13	21.00	-1.68	0.09
FCI-SCM- tot	6.85	5.57	8.51	4.96	28.00	-1.06	0.29
FCSI-SCM	-2.22	8.40	-0.38	42.14	37.00	-0.26	0.79
UT-R	11.59	8.34	11.29	7.11	40.00	0.00	1.00
UT-L	9.46	6.17	8.13	4.98	39.00	0.09	0.93
UT-tot	10.52	7.11	9.71	5.03	38.00	-0.18	0.86
AsI -UT	6.77	16.92	11.14	33.55	35.00	-0.44	0.66
FCI-UT-R	2.29	1.53	1.79	1.29	29.00	0.97	0.33
FCI-UT-L	1.48	0.82	1.55	0.33	37.00	-0.26	0.79
FCI-UT-tot	1.36	0.47	1.45	0.61	38.00	-0.18	0.86
FCSI-UT	16.72	36.52	-0.26	26.18	31.00	0.79	0.43
**MVC-TA-R**	91.66	33.41	97.90	41.49	37.00	-0.26	0.79
**MVC-TA-L**	96.76	24.98	102.95	42.03	40.00	0.00	1.00
**MVC-TA-tot**	93.89	28.59	99.79	41.78	39.00	-0.09	0.93
**MVC-MM-R**	71.34	38.46	99.97	50.88	27.00	-1.15	0.25
**MVC-MM-L**	72.89	36.76	91.00	49.57	34.00	-0.53	0.60
**MVC-MM-tot**	71.65	36.81	95.07	50.46	31.00	-0.79	0.43
**MVC-SCM-R**	60.89	32.20	226.93	323.05	14.00	-2.30	0.02*
**MVC-SCM-L**	69.87	39.00	144.05	101.67	16.00	-2.12	0.03*
**MVC-SCM-tot**	65.14	34.83	175.11	184.71	12.00	-2.47	0.01*
**MVC-UT-R**	77.97	31.44	78.01	43.63	35.00	0.44	0.66
**MVC-UT-L**	83.15	27.31	84.59	29.93	36.00	-0.35	0.72
**MVC-UT-tot**	80.85	24.64	80.75	34.40	38.00	0.18	0.86

AcI— activity index; AEO— abdominal external oblique muscle; AsI — asymmetry index; BB — biceps brachii muscle; ES—effect size; FCAI— Functional Clenching Activity Index; FCI — Functional Clenching Index; FCoI— Functional Contraction Index; FCoSI— Functional Contraction Symmetry Index; FCSI— Functional Clenching Symmetry Index; L— left side; MM — the superficial part of the masseter muscle; MVC— maximum voluntary contraction; n— individuals in the sample; POC— percentage overlapping coefficient; R— right side; RA-lo — the lower part of the rectus abdominis muscle; RA-up— the upper part of the rectus abdominis muscle; SCM — the middle part of the sternocleidomastoid muscle; SD — standard deviation; TA — the anterior part of the temporalis muscle; TC— torque coefficient; U— the difference between the two rank totals; UT— the upper part of the trapezius muscle; Z— the Mann-Whitney U test; * significant difference.

Table S5. Comparison of bioelectrical activity findings of masticatory and cervical spine muscles in emmetropic subjects.

		**Without Paralysis of Accommodation**		**Accommodation Paralysis in The Right Eye**		**Accommodation Paralysis in The Left Eye**		**H**	**p**
		Mean	SD	Mean	SD	Mean	SD		
**Rest**	TA-R	5.39	2.74	4.94	1.79	4.99	2.46	0.39	0.82
	TA-L	8.64	6.71	7.10	3.48	6.47	3.47	0.32	0.85
	TA-tot	7.02	3.99	6.02	1.73	5.73	2.24	0.17	0.91
	AsI -TA	-15.86	30.94	-14.30	28.76	-9.34	30.08	0.28	0.86
	MM-R	3.91	2.91	3.91	3.49	4.00	2.49	0.11	0.94
	MM-L	3.77	3.83	4.73	4.01	3.99	2.45	1.02	0.59
	MM-tot	3.84	3.29	4.32	3.73	4.00	2.28	0.85	0.65
	AsI -MM	9.67	37.37	-10.41	13.24	-1.86	24.63	3.09	0.21
	AcI-R	-18.14	38.49	-18.12	33.52	-13.11	28.54	0.18	0.91
	AcI-L	-36.77	41.07	-22.12	33.63	-20.94	32.47	0.71	0.70
	AcI-tot	-29.78	36.33	-22.52	31.79	-18.65	25.53	1.79	0.40
	POC	92.98	45.83	82.55	33.96	94.08	37.71	0.34	0.84
	TC	-337.67	634.41	-134.22	449.11	-149.89	433.21	0.60	0.74
	SCM-R	3.66	1.41	3.47	1.52	4.00	1.75	0.24	0.88
	SCM-L	3.45	1.39	4.22	3.37	3.29	0.80	0.04	0.97
	SCM-tot	3.55	0.86	3.84	1.93	3.64	1.18	0.17	0.91
	AsI -SCM	3.52	26.76	-3.86	25.84	6.82	16.34	1.33	0.51
	UT-R	8.33	7.43	5.39	5.17	6.80	5.99	0.87	0.64
	UT-L	10.14	12.96	5.40	3.47	28.01	64.22	1.44	0.48
	UT-tot	9.24	9.45	5.39	3.85	17.40	31.35	1.07	0.58
	AsI -UT	-5.50	44.04	-6.07	32.02	-13.03	39.65	0.28	0.86
**Clenching in The Intercuspal Position**	TA-R	178.86	81.31	168.65	79.71	188.30	82.96	0.46	0.79
	TA-L	167.54	88.51	161.52	86.57	186.69	88.70	0.65	0.72
	TA-tot	173.20	77.02	165.09	75.66	187.49	76.59	0.40	0.82
	AsI -TA	3.16	16.44	1.72	18.56	1.08	19.78	0.07	0.97
	FCI-TA-R	40.12	24.78	37.08	19.83	50.20	36.00	0.52	0.77
	FCI-TA-L	30.80	28.34	29.22	29.05	46.40	47.36	0.69	0.70
	FCI-TA -tot	31.77	23.78	30.39	22.62	44.35	39.29	0.60	0.73
	FCSI-TA	17.16	35.12	15.03	31.78	9.77	36.01	0.24	0.88
	MM-R	220.49	162.32	225.16	179.85	236.27	187.08	0.03	0.98
	MM-L	189.96	123.40	186.74	124.11	199.55	130.05	0.10	0.95
	MM-tot	205.23	138.39	205.95	148.79	217.91	155.30	0.07	0.96
	AsI -MM	2.78	17.87	4.30	15.90	3.69	15.61	0.05	0.97
	AcI-R	-1.63	29.26	0.94	29.39	-2.13	31.27	0.20	0.90
	AcI-L	-1.34	24.69	-1.19	26.52	-4.04	31.03	0.10	0.95
	AcI- tot	-1.39	23.97	-0.26	24.99	-3.42	27.85	0.10	0.95
	FCI-MM-R	89.03	91.11	89.59	88.90	92.22	94.61	0.00	1.00
	FCI-MM-L	1869.68	5399.22	55.94	41.27	59.47	43.28	0.53	0.76
	FCI-MM-tot	81.57	69.38	67.52	53.07	70.85	60.97	0.17	0.91
	FCSI-MM	-9.69	43.48	14.14	24.72	5.57	33.02	2.30	0.31
	FCAI-R	15.18	41.13	17.50	43.76	10.90	33.53	0.13	0.93
	FCAI-L	31.13	51.14	18.58	46.78	14.21	38.64	0.34	0.84

	FCAI-tot	25.93	43.97	20.62	43.40	13.94	30.84	0.69	0.70
	POC	109.84	22.45	109.61	23.44	106.91	21.51	0.11	0.94
	TC	-1921.48	11549.16	-3128.33	11170.30	-3510.74	12302.01	0.45	0.79
	SCM-R	11.73	7.92	13.03	12.47	14.78	12.90	0.17	0.91
	SCM-L	12.61	12.66	16.09	18.54	16.70	19.16	0.24	0.88
	SCM-tot	12.17	10.00	14.56	15.37	15.74	15.73	0.02	0.99
	AsI -SCM	1.25	15.73	-5.36	19.20	-1.02	17.53	0.65	0.72
	FCI-SCM-R	3.46	2.38	4.00	4.13	3.87	3.66	0.17	0.91
	FCI-SCM-L	4.79	6.18	5.12	6.86	4.82	5.12	0.22	0.89
	FCI-SCM-tot	3.93	3.94	4.50	5.46	4.32	4.43	0.07	0.96
	FCSI-SCM	-2.39	27.06	-0.76	19.04	-7.72	13.78	0.26	0.87
	UT-R	7.67	5.95	10.28	9.54	8.58	7.08	0.17	0.91
	UT-L	9.97	9.39	6.35	3.73	7.26	4.23	0.91	0.63
	UT-tot	8.82	6.32	8.31	6.05	7.92	5.14	0.05	0.97
	ASI-UT	-12.79	32.67	8.65	27.71	1.79	32.24	3.56	0.16
	FCI-UT-R	1.29	1.14	2.54	2.71	1.42	0.54	2.40	0.30
	FCI-UT-L	1.51	1.22	1.30	0.64	1.19	0.69	0.03	0.98
	FCI-UT-tot	1.10	0.34	1.73	1.49	1.17	0.53	0.15	0.92
	FCSI-UT	7.88	48.49	15.65	25.52	10.39	38.79	1.03	0.59
	TA-R	179.70	46.86	190.23	70.03	206.81	62.67	0.91	0.63
	TA-L	171.31	65.77	179.31	91.68	190.63	72.09	0.27	0.87
	TA-tot	175.51	46.69	184.77	75.82	198.72	59.45	0.96	0.62
	AsI -TA	4.14	19.12	5.76	17.90	5.34	17.42	0.10	0.95
	FCI-TA-R	39.60	18.38	41.80	17.23	51.39	29.41	1.06	0.58
	FCI-TA-L	32.24	23.91	33.50	29.23	47.72	46.43	0.52	0.77
	FCI-TA -tot	33.06	19.44	34.94	22.02	46.62	37.29	0.45	0.79
	FCSI-TA	18.46	31.00	19.08	30.49	13.90	29.69	0.22	0.89
	MM-R	243.19	119.93	290.44	185.96	304.64	159.72	1.03	0.59
	MM-L	218.81	78.91	257.19	138.20	258.93	117.29	0.47	0.79
	MM-tot	231.00	96.87	273.81	157.72	281.79	133.57	0.87	0.64
**Clenching on Dental Cotton Rollers**	AsI -MM	2.81	14.60	3.86	17.75	5.65	17.31	0.47	0.79
	AcI-R	10.92	22.73	14.28	25.11	13.77	25.27	0.10	0.95
	AcI-L	12.50	17.86	16.40	21.49	13.67	22.56	0.33	0.84
	AcI- tot	11.41	15.30	15.39	19.82	13.82	20.21	0.09	0.95
	FCI-MM-R	88.98	73.72	109.62	89.82	113.96	95.55	0.13	0.93
	FCI-MM-L	3291.50	9653.32	71.59	40.86	76.54	32.95	0.15	0.92
	FCI-MM-tot	90.11	68.10	85.54	55.99	87.93	56.05	0.00	0.99
	FCSI-MM	-6.97	40.75	13.89	24.92	7.95	36.07	2.00	0.36
	FCAI-R	26.93	35.05	28.44	39.23	25.75	28.25	0.74	0.96
	FCAI-L	42.12	46.69	32.79	37.97	30.30	37.02	0.55	0.75
	FCAI-tot	37.60	38.42	33.53	34.89	30.32	24.32	0.52	0.77
	POC	110.09	23.08	113.99	28.40	115.22	25.76	0.34	0.84
	TC	-1597.41	9833.20	-2233.70	11314.51	-2953.70	10727.11	0.26	0.87
	SCM-R	32.64	51.80	17.52	11.15	23.18	14.18	1.01	0.60
	SCM-L	15.47	9.79	21.47	20.50	23.19	21.48	0.91	0.63
	SCM-tot	24.05	26.47	19.49	15.13	23.18	17.51	0.45	0.79
	AsI-SCM	11.56	33.94	-3.44	20.47	4.65	14.05	1.14	0.56

FCI-SCM-R	9.50	14.41	5.21	3.31	6.76	5.31	0.53	0.76
FCI-SCM-L	5.45	4.93	6.84	7.60	7.03	5.88	1.19	0.55
FCI-SCM-tot	7.39	8.12	5.92	5.46	6.85	5.57	0.67	0.71
FCSI-SCM	8.08	41.04	0.10	26.70	-2.22	8.40	0.07	0.96
UT-R	10.27	7.09	9.97	8.36	11.59	8.34	0.18	0.91
UT-L	7.25	2.91	7.41	4.18	9.46	6.17	0.30	0.86
UT-tot	8.76	4.38	8.69	5.66	10.52	7.11	0.32	0.85
AsI-UT	10.48	25.49	3.36	24.10	6.77	16.92	0.52	0.77
FCI-UT-R	2.06	1.93	2.75	3.15	2.29	1.53	0.74	0.68
FCI-UT-L	1.44	1.11	1.51	0.64	1.48	0.82	0.26	0.87
FCI-UT-tot	1.25	0.66	1.14	0.34	1.36	0.47	1.27	0.52
FCSI-UT	15.30	40.19	9.88	28.59	16.72	36.52	0.22	0.89
**MVC-TA-R**	99.03	38.02	89.93	38.46	91.66	33.41	0.28	0.86
**MVC-TA-L**	98.33	32.12	94.72	33.43	96.76	24.98	0.17	0.91
**MVC-TA-tot**	97.81	34.47	91.91	35.62	93.89	28.59	0.09	0.95
**MVC-MM-R**	87.21	52.65	72.15	37.63	71.34	38.46	0.46	0.79
**MVC-MM-L**	82.97	48.53	70.94	34.79	72.89	36.76	0.08	0.96
**MVC-MM-tot**	84.76	49.10	71.02	34.99	71.65	36.81	0.40	0.82
**MVC-SCM-R**	66.41	36.01	67.72	22.09	60.89	32.20	0.55	0.75
**MVC-SCM-L**	74.78	26.69	72.34	28.23	69.87	39.00	0.81	0.66
**MVC-SCM-tot**	65.07	31.85	68.92	22.22	65.14	34.83	0.43	0.80
**MVC-UT-R**	77.76	32.93	99.89	33.11	77.97	31.44	1.73	0.42
**MVC-UT-L**	151.93	168.84	87.12	20.03	83.15	27.31	1.56	0.45
**MVC-UT-tot**	107.84	80.67	94.00	23.70	80.85	24.64	1.27	0.52

AcI— activity index; AEO— abdominal external oblique muscle; AsI— asymmetry index; BB — biceps brachii muscle; ES—effect size; FCAI— Functional Clenching Activity Index; FCI — Functional Clenching Index; FCoI— Functional Contraction Index; FCoSI— Functional Contraction Symmetry Index; FCSI— Functional Clenching Symmetry Index; L— left side; MM — the superficial part of the masseter muscle; MVC— maximum voluntary contraction; n— individuals in the sample; POC— percentage overlapping coefficient; R— right side; RA-lo — the lower part of the rectus abdominis muscle; RA-up— the upper part of the rectus abdominis muscle; SCM — the middle part of the sternocleidomastoid muscle; SD — standard deviation; TA — the anterior part of the temporalis muscle; TC— torque coefficient; U— the difference between the two rank totals; UT— the upper part of the trapezius muscle H— the Kruskal-Wallis test;

Table S6. Comparison of bioelectrical activity findings of masticatory and cervical spine muscles in myopic subjects.

		Without Paralysis of Accommodation		Accommodation Paralysis in The Right Eye		Accommodation Paralysis in The Left Eye		H	p
		Mean	SD	Mean	SD	Mean	SD		
**Rest**	TA-R	6.49	5.38	7.31	5.91	7.03	5.35	0.74	0.96
	TA-L	7.38	4.21	7.02	4.50	7.11	3.60	0.05	0.97
	TA-tot	6.93	4.54	7.17	5.06	7.07	4.16	0.12	0.93
	ASI-TA	-13.02	31.95	-5.23	24.10	-7.22	25.10	0.18	0.91
	MM-R	3.97	3.39	3.37	1.63	3.21	2.05	0.26	0.87
	MM-L	5.86	8.66	6.31	9.96	6.39	11.12	0.87	0.64
	MM-tot	4.91	5.95	4.84	5.52	4.80	6.44	1.07	0.58
	AsI-MM	-7.00	22.67	-9.00	33.79	-10.60	27.35	0.24	0.88
	AcI-R	-18.09	39.86	-24.37	35.68	-29.20	35.68	0.11	0.94
	AcI-L	-25.05	38.65	-20.27	39.08	-26.99	39.13	0.27	0.87
	AcI-tot	-23.96	34.16	-20.82	30.51	-27.00	35.37	0.34	0.84
	POC	86.25	36.61	88.89	27.59	89.24	38.89	0.04	0.97
	TC	99.56	620.33	323.44	1133.55	310.22	1198.30	2.26	0.95
	SCM-R	2.89	1.61	4.24	3.81	4.45	6.60	2.25	0.32
	SCM-L	3.01	1.23	3.05	1.04	2.66	0.63	0.29	0.86
	SCM-tot	2.95	0.98	3.64	1.95	3.56	3.27	1.51	0.47
	AsI-SCM	-3.92	27.43	6.70	26.14	1.65	33.94	0.77	0.68
	UT-R	5.31	3.43	6.64	6.64	8.36	6.94	0.77	0.68
	UT-L	3.81	1.22	4.88	3.07	5.25	2.94	1.63	0.44
	UT-tot	4.56	1.70	5.76	3.77	6.81	4.63	1.00	0.60
	AsI -UT	6.42	39.22	1.46	43.23	10.71	36.98	0.07	0.96
**Clenching in The Intercuspal Position**	TA-R	142.63	43.19	145.50	45.88	155.70	53.57	0.22	0.89
	TA-L	150.33	52.85	130.69	69.60	158.86	59.84	0.62	0.73
	TA-tot	146.48	43.56	138.09	44.35	157.28	47.54	0.47	0.79
	AsI -TA	-2.73	15.28	8.73	36.70	-1.25	18.92	0.07	0.96
	FCI-TA-R	41.58	31.35	38.88	30.06	36.78	23.77	0.05	0.97
	FCI-TA-L	30.53	23.97	26.68	21.59	29.89	21.32	0.27	0.87
	FCI-TA -tot	31.51	21.62	30.23	20.49	31.77	20.78	0.05	0.97
	FCSI-TA	10.38	34.45	12.57	41.76	5.63	27.37	0.10	0.95
	MM-R	297.11	204.74	302.33	204.12	326.11	239.26	0.07	0.96
	MM-L	261.62	191.41	284.64	208.67	276.94	221.89	0.11	0.94
	MM-tot	279.37	196.66	293.48	205.02	301.53	228.79	0.07	0.96
	AsI -MM	3.89	14.50	2.65	11.26	7.92	13.71	0.80	0.67
	AcI-R	22.78	34.00	23.55	31.70	23.28	32.19	0.03	0.98
	AcI-L	16.09	27.94	29.34	37.79	14.09	30.48	1.23	0.54
	AcI- tot	19.46	29.78	24.57	29.37	18.69	29.89	0.32	0.85
	FCI-MM-R	128.46	120.79	126.61	122.52	146.85	144.60	0.17	0.91
	FCI-MM-L	90.51	89.03	87.44	79.95	109.72	119.72	0.05	0.97
	FCI-MM-tot	104.47	100.56	97.55	88.19	123.98	132.11	0.03	0.98
	FCSI-MM	10.49	31.84	11.84	35.92	16.91	33.05	0.09	0.95
	FCAI-R	33.42	45.60	40.32	40.75	44.88	38.82	0.39	0.82
	FCAI-L	39.47	33.91	45.49	34.74	37.77	38.68	0.32	0.85

	FCAI-tot	38.81	35.48	41.74	28.82	40.87	34.05	0.09	0.95
	POC	106.75	22.74	116.49	44.67	112.58	28.64	0.17	0.91
	TC	-4318.59	6235.52	-288.00	8012.85	-5234.44	7683.73	1.32	0.51
	SCM-R	18.98	12.58	37.80	57.72	59.02	107.21	0.96	0.61
	SCM-L	27.54	24.85	19.96	9.49	28.06	17.40	0.61	0.73
	SCM-tot	23.26	16.55	28.88	29.41	43.54	58.20	0.46	0.79
	AsI -SCM	-13.89	22.08	6.14	34.81	3.61	33.82	2.21	0.33
	FCI-SCM-R	8.32	7.47	14.14	23.72	21.53	35.31	0.38	0.82
	FCI-SCM-L	11.07	12.23	7.44	5.20	11.09	7.25	1.10	0.57
	FCI-SCM-tot	8.44	6.20	10.14	11.58	16.26	21.16	0.47	0.79
	FCSI-SCM	-7.45	33.26	-1.84	42.26	1.40	41.22	0.32	0.85
	UT-R	6.32	5.04	8.72	7.70	7.84	5.04	1.12	0.57
	UT-L	4.60	1.23	6.75	3.39	6.01	2.73	2.26	0.32
	UT-tot	5.46	2.54	7.73	5.23	6.93	2.65	1.51	0.47
	AsI -UT	2.05	41.52	-2.53	33.79	4.23	41.35	0.07	0.96
	FCI-UT-R	1.16	0.38	1.34	0.38	1.09	0.36	0.91	0.63
	FCI-UT-L	1.31	0.56	1.57	0.91	1.29	0.47	0.74	0.68
	FCI-UT-tot	1.23	0.42	1.34	0.39	1.18	0.36	0.90	0.63
	FCSI-UT	-5.25	16.88	-3.89	22.41	-7.20	16.80	0.27	0.87
**Clenching on Dental Cotton Rollers**	TA-R	140.31	56.45	162.56	65.42	171.46	65.71	1.15	0.56
	TA-L	140.89	44.62	153.40	43.72	160.15	47.38	2.18	0.33
	TA-tot	140.60	36.67	157.98	44.34	165.80	41.62	2.00	0.36
	AsI-TA	-1.82	23.31	0.64	19.91	1.43	21.76	0.10	0.95
	FCI-TA-R	40.29	32.37	43.93	39.66	41.01	32.14	0.07	0.96
	FCI-TA-L	25.99	17.14	30.98	19.97	28.44	15.65	0.40	0.82
	FCI-TA -tot	29.04	19.04	35.04	26.24	32.41	19.81	0.22	0.89
	FCSI-TA	10.76	35.17	5.79	27.10	7.73	29.97	0.03	0.98
	MM-R	273.97	116.03	307.34	140.65	309.06	150.30	0.68	0.71
	MM-L	258.29	109.04	290.20	147.52	287.96	148.07	0.07	0.96
	MM-tot	266.13	104.66	298.77	138.10	298.51	143.68	0.17	0.91
	AsI-MM	0.71	19.03	1.60	18.06	1.93	18.00	0.04	0.97
	AcI-R	28.42	28.68	26.64	26.74	23.74	27.73	0.32	0.85
	AcI-L	26.90	17.66	26.24	17.83	23.34	22.32	0.38	0.82
	AcI- tot	27.63	19.49	26.44	20.38	23.41	22.72	0.30	0.86
	FCI-MM-R	107.09	74.80	114.68	76.12	127.23	98.96	0.15	0.92
	FCI-MM-L	85.99	50.31	92.48	60.61	108.20	87.51	0.10	0.95
	FCI-MM-tot	92.54	59.53	93.49	53.29	112.28	91.00	0.18	0.91
	FCSI-MM	7.46	33.44	9.69	41.43	10.94	35.63	0.01	0.99
	FCAI-R	40.69	37.12	44.23	34.20	46.63	34.66	0.20	0.90
	FCAI-L	47.49	37.30	43.61	34.87	45.41	39.42	0.36	0.83
	FCAI-tot	46.81	30.18	44.00	25.73	45.93	29.67	0.40	0.82
	POC	106.72	36.07	109.78	39.49	110.21	39.53	0.07	0.96
	TC	-1625.56	8668.20	-797.78	6085.07	-978.52	8422.87	0.10	0.95
	SCM-R	17.98	10.95	19.18	7.45	23.51	16.70	0.77	0.67
	SCM-L	18.26	13.04	19.30	11.26	20.88	8.23	1.23	0.54
	SCM-tot	18.12	11.54	19.24	8.70	22.20	9.39	2.32	0.31
	AsI-SCM	-0.44	19.87	2.26	20.94	-0.39	29.02	0.81	0.66

FCI-SCM-R	8.44	8.76	6.23	3.77	10.56	9.25	1.06	0.58
FCI-SCM-L	6.66	5.30	6.59	3.18	7.73	2.13	2.73	0.25
FCI-SCM-tot	7.14	6.49	6.13	3.30	8.51	4.96	1.17	0.55
FCSI-SCM	2.83	25.05	-5.20	32.09	-0.38	42.14	0.26	0.87
UT-R	10.76	6.72	11.09	7.70	11.29	7.11	0.30	0.98
UT-L	7.18	4.42	7.79	5.40	8.13	4.98	0.73	0.69
UT-tot	8.97	4.85	9.44	5.77	9.71	5.03	0.10	0.95
AsI-UT	15.08	38.46	11.20	37.39	11.14	33.55	0.17	0.91
FCI-UT-R	2.77	2.70	2.67	2.62	1.79	1.29	0.22	0.89
FCI-UT-L	1.78	0.78	1.73	1.13	1.55	0.33	0.96	0.61
FCI-UT-tot	1.88	1.21	1.45	0.80	1.45	0.61	0.77	0.67
FCSI-UT	8.38	28.21	5.72	42.58	-0.26	26.18	0.68	0.71
**MVC-TA-R**	110.88	43.36	98.53	42.11	97.90	41.49	0.62	0.73
**MVC-TA-L**	113.01	49.11	92.29	53.20	102.95	42.03	0.95	0.62
**MVC-TA-tot**	109.56	44.45	94.08	44.38	99.79	41.78	2.12	0.34
**MVC-MM-R**	103.40	61.85	95.90	54.80	99.97	50.88	0.35	0.83
**MVC-MM-L**	97.78	57.83	97.02	57.15	91.00	49.57	0.02	0.99
**MVC-MM-tot**	99.95	59.68	95.27	55.20	95.07	50.46	0.03	0.98
**MVC-SCM-R**	107.88	47.35	174.99	228.12	226.93	323.05	0.23	0.89
**MVC-SCM-L**	217.74	307.82	122.75	77.23	144.05	101.67	0.17	0.91
**MVC-SCM-tot**	145.51	119.07	147.60	122.04	175.11	184.71	0.14	0.93
**MVC-UT-R**	70.34	47.94	92.62	65.06	78.01	43.63	0.44	0.80
**MVC-UT-L**	90.29	66.86	116.61	85.15	84.59	29.93	0.74	0.68
**MVC-UT-tot**	77.83	57.72	95.15	58.48	80.75	34.40	0.77	0.67

AcI— activity index; AEO— abdominal external oblique muscle; AsI— asymmetry index; BB — biceps brachii muscle; ES—effect size; FCAI— Functional Clenching Activity Index; FCI — Functional Clenching Index; FCoI— Functional Contraction Index; FCoSI— Functional Contraction Symmetry Index; FCSI— Functional Clenching Symmetry Index; L— left side; MM — the superficial part of the masseter muscle; MVC— maximum voluntary contraction; n— individuals in the sample; POC— percentage overlapping coefficient; R— right side; RA-lo — the lower part of the rectus abdominis muscle; RA-up— the upper part of the rectus abdominis muscle; SCM — the middle part of the sternocleidomastoid muscle; SD — standard deviation; TA — the anterior part of the temporalis muscle; TC— torque coefficient; U— the difference between the two rank totals; UT— the upper part of the trapezius muscle; H— the Kruskal-Wallis test.

Table S7. Comparison of bioelectrical activity findings of postural muscles and the arm muscles in emmetropic subjects and myopic subjects (Without Paralysis of Accommodation).

		**Emmetropic Subjects**		**Myopic Subjects**				
		Mean	SD	Mean	SD	U	Z	P
**Lying Position**	BB-R	4.11	2.00	3.76	2.38	23.50	-0.79	0.43
	BB-L	4.68	6.52	2.78	1.37	30.00	0.11	0.92
	BB-tot	4.39	3.90	3.20	1.56	29.00	-0.21	0.83
	BB-R-%	58.86	18.25	51.87	21.41	30.00	-0.11	0.92
	BB-L-%	41.14	18.25	39.58	18.39	30.00	0.11	0.92
	AsI-BB	17.71	36.49	16.24	31.98	30.00	-0.11	0.92
	RA-up-R	7.38	3.21	7.24	3.16	26.00	-0.53	0.60
	RA-up-L	6.52	3.26	6.31	2.75	28.00	-0.32	0.75
	RA-lo-R	4.91	1.73	3.52	0.87	14.50	-1.75	0.08
	RA-lo-L	10.81	9.67	4.52	2.47	17.00	-1.48	0.14
	RA-up-tot	10.64	4.48	10.37	3.42	27.00	-0.42	0.67
	RA-lo-tot	10.32	5.19	5.68	1.67	12.50	-1.96	0.05
	RA-up-R-%	26.61	9.07	31.19	13.44	18.00	1.38	0.17
	RA-up-L-%	22.99	7.58	26.73	9.01	19.00	1.27	0.20
	RA-lo-R-%	18.73	7.96	17.03	5.45	31.00	0.00	1.00
	RA-lo-L-%	31.67	18.98	17.83	5.30	22.00	-0.95	0.34
	AsI-RA-up	6.18	19.03	10.17	24.80	31.00	0.00	1.00
	AsI-RA-lo	-17.69	40.54	2.74	25.31	26.00	0.53	0.60
	AEO-R	6.71	5.16	3.46	2.30	7.00	-2.54	0.01*
	AEO-L	5.24	4.22	5.35	2.83	29.00	0.21	0.83
	AEO-tot	5.97	4.59	3.99	1.42	17.00	-1.48	0.14
	AEO-R-%	55.24	11.06	36.17	10.05	8.00	-2.43	0.01*
	AEO-L-%	44.76	11.06	56.82	16.33	8.00	2.43	0.01*
	AsI-AEO	10.48	22.12	-15.16	29.01	8.00	-2.43	0.01*
**Standing Position**	BB-R	2.45	0.71	2.23	0.85	30.00	-0.11	0.92
	BB-L	1.77	0.98	1.14	0.37	17.50	-1.43	0.15
	BB-tot	2.11	0.62	1.65	0.57	23.00	-0.85	0.40
	BB-R-%	59.10	12.02	58.70	20.27	20.00	1.16	0.24
	BB-L-%	40.90	12.02	32.20	11.63	20.00	-1.16	0.24
	AsI-BB	18.20	24.05	29.90	17.77	20.00	1.16	0.24
	RA-up-R	8.72	6.10	7.07	3.51	28.00	-0.32	0.75
	RA-up-L	7.46	3.85	7.17	3.22	29.00	0.21	0.83
	RA-lo-R	4.14	0.59	4.80	2.63	21.00	1.06	0.29
	RA-lo-L	5.81	8.47	3.69	2.75	27.00	-0.42	0.67
	RA-up-tot	12.44	7.92	10.54	5.16	29.00	-0.21	0.83
	RA-lo-tot	7.05	4.58	6.62	3.91	27.00	0.42	0.67
	RA-up-R-%	32.73	11.05	27.25	8.41	25.00	-0.64	0.53
	RA-up-L-%	29.52	7.00	28.50	8.70	29.00	0.21	0.83
	RA-lo-R-%	18.55	7.42	20.85	9.36	19.00	1.27	0.20
	RA-lo-L-%	19.20	15.99	15.20	6.22	29.00	0.21	0.83

	ASI-RA-up	3.71	15.15	2.05	13.31	27.00	-0.42	0.67
	ASI-RA-lo	6.00	32.53	16.68	16.75	29.00	0.21	0.83
	AEO-R	9.04	5.57	5.89	4.27	17.00	-1.48	0.14
	AEO-L	7.59	3.95	5.82	2.79	22.50	-0.90	0.37
	AEO-tot	8.31	4.54	5.81	2.65	19.00	-1.27	0.20
	AEO-R-%	52.53	8.91	42.66	18.50	19.00	-1.27	0.20
	AEO-L-%	47.47	8.91	47.40	19.90	19.00	1.27	0.20
	AsI-AEO	5.05	17.83	-2.18	27.68	19.00	-1.27	0.20
**Sitting Position**	BB-R	2.23	0.69	2.09	0.88	29.00	0.21	0.83
	BB-L	1.46	0.89	1.20	0.49	31.50	-0.05	0.96
	BB-tot	1.84	0.56	1.62	0.63	31.00	0.00	1.00
	BB-R-%	61.52	13.33	57.27	20.88	30.00	0.11	0.92
	BB-L-%	38.48	13.33	32.82	12.62	30.00	-0.11	0.92
	AsI-BB	23.04	26.65	27.03	17.71	30.00	0.11	0.92
	RA-up-P-R	6.60	4.20	5.13	2.15	29.00	-0.21	0.83
	RA-up-L	7.72	7.26	6.20	2.31	29.00	0.21	0.83
	RA-lo-R	3.73	1.16	2.93	0.81	21.00	-1.06	0.29
	RA-lo-L	6.18	11.77	2.81	1.88	25.00	-0.64	0.53
	RA-up-tot	10.46	5.66	8.19	3.27	26.00	-0.53	0.60
	RA-lo-tot	6.81	6.40	4.32	0.75	25.00	-0.64	0.53
	RA-up-R-%	32.28	13.45	28.17	9.26	28.00	-0.32	0.75
	RA-up-L-%	30.79	5.43	33.33	8.92	15.00	1.69	0.09
	RA-lo-R-%	19.38	8.05	17.91	9.16	29.00	-0.21	0.83
	RA-lo-L-%	17.56	13.23	14.31	4.79	29.00	-0.21	0.83
	AsI-RA-up	-1.58	30.28	-1.53	16.65	21.00	-1.06	0.29
	AsI-RA-lo	11.18	36.31	19.97	17.09	31.00	0.00	1.00
	AEO-R	3.14	0.73	2.40	1.01	17.50	-1.43	0.15
	AEO-L	3.63	1.11	3.83	1.88	23.00	0.85	0.40
	AEO-tot	3.39	0.83	3.11	1.34	30.00	-0.11	0.92
	AEO-R-%	46.86	6.42	35.16	14.22	19.00	-1.27	0.20
	AEO-L-%	53.14	6.42	53.55	20.97	19.00	1.27	0.20
	AsI-AEO	-6.28	12.83	-17.19	18.06	19.00	-1.27	0.20
	BB-R	477.53	359.67	825.12	471.41	11.00	2.12	0.03*
**Maximum Voluntary Contraction**	BB-L	618.89	390.92	858.94	384.31	15.00	1.69	0.09
	BB-tot	548.21	329.99	836.57	381.18	14.00	1.80	0.07
	BB-R-%	41.85	12.78	43.23	17.80	21.00	1.06	0.29
	BB-L-%	58.15	12.78	47.21	18.98	21.00	-1.06	0.29
	AsI-BB	-16.31	25.56	-1.04	26.82	21.00	1.06	0.29
	RA-up-R	97.55	78.76	126.78	66.40	23.00	0.85	0.40
	RA-up-L	109.19	80.82	136.47	90.76	22.00	0.95	0.34
	RA-lo-R	69.26	72.09	131.72	95.12	18.00	1.38	0.17
	RA-lo-L	300.63	601.07	150.06	73.42	18.00	1.38	0.17
	RA-up-tot	152.14	117.79	194.79	106.93	23.00	0.85	0.40
	RA-lo-tot	219.57	315.55	206.68	126.19	23.00	0.85	0.40
	RA-up-R-%	26.52	12.06	22.89	7.23	27.00	-0.42	0.67
	RA-up-L-%	28.36	11.14	24.07	9.94	24.00	-0.74	0.46
	RA-lo-R-%	17.09	7.28	20.41	10.83	23.00	0.85	0.40
	RA-lo-L-%	28.03	26.27	24.04	7.33	21.00	1.06	0.29

AsI-RA-up	-6.91	13.62	0.89	14.20	23.00	0.85	0.40
AsI-RA-lo	-8.92	37.90	-6.21	21.45	27.00	-0.42	0.67
AEO-R	65.49	71.55	111.93	127.79	22.00	0.95	0.34
AEO-L	87.22	114.21	162.56	89.60	18.00	1.38	0.17
AEO-tot	76.35	92.78	136.96	96.70	18.00	1.38	0.17
AEO-R-%	46.62	5.27	36.43	17.48	25.00	-0.64	0.53
AEO-L-%	53.38	5.27	53.51	22.40	25.00	0.64	0.53
AsI-AEO	-6.76	10.54	-14.63	30.82	25.00	-0.64	0.53
**FCoI-BB-R**	127.63	107.12	260.56	141.83	9.00	2.33	0.02*
**FCoI-BB-L**	321.70	337.60	404.66	146.74	15.00	1.69	0.09
**FCoI-RA-up-R**	15.24	14.80	22.04	15.58	18.00	1.38	0.17
**FCoI-RA-up-L**	19.83	18.39	26.96	19.54	16.00	1.59	0.11
**FCoI-RA-lo-R**	18.06	26.95	42.40	31.61	12.00	2.01	0.04*
**FCoI-RA-lo-L**	83.55	164.86	60.46	50.35	14.00	1.80	0.07
**FCoI-AEO-R**	14.77	17.92	39.21	41.32	13.00	1.91	0.06
**FCoI-AEO-L**	28.13	43.03	40.94	24.59	19.00	1.27	0.20
**FCoSI-BB**	-32.27	36.44	-11.79	35.85	22.00	0.95	0.34
**FCoSI-RA-up**	-13.02	19.41	-6.60	25.22	27.00	0.42	0.67
**FCoSI-RA-lo**	9.19	57.75	-2.12	34.13	23.00	-0.85	0.40
**FCoSI-AEO**	-16.18	28.00	8.97	39.04	18.00	1.38	0.17

AcI— activity index; AEO— abdominal external oblique muscle; AsI— asymmetry index; BB — biceps brachii muscle; ES—effect size; FCAI— Functional Clenching Activity Index; FCI — Functional Clenching Index; FCoI— Functional Contraction Index; FCoSI— Functional Contraction Symmetry Index; FCSI— Functional Clenching Symmetry Index; L— left side; MM — the superficial part of the masseter muscle; MVC— maximum voluntary contraction; n— individuals in the sample; POC— percentage overlapping coefficient; R— right side; RA-lo — the lower part of the rectus abdominis muscle; RA-up— the upper part of the rectus abdominis muscle; SCM — the middle part of the sternocleidomastoid muscle; SD — standard deviation; TA — the anterior part of the temporalis muscle; TC— torque coefficient; U— the difference between the two rank totals; UT— the upper part of the trapezius muscle; Z— the Mann-Whitney U test; * significant difference.

Table S8. Comparison of bioelectrical activity findings of postural muscles and the arm muscles in emmetropic subjects and myopic subjects (Accommodation Paralysis in The Right Eye).

		Emmetropic Subjects		Myopic Subjects		U	Z	P
		Mean	SD	Mean	SD			
**Lying Position**	BB-R	3.80	1.36	3.68	2.02	28.50	-0.26	0.79
	BB-L	4.45	5.01	3.52	4.23	30.00	0.11	0.92
	BB-tot	4.12	2.97	3.56	2.95	30.00	-0.11	0.92
	BB-R-%	57.18	19.50	52.49	18.34	28.00	-0.32	0.75
	BB-L-%	42.82	19.50	39.98	15.96	28.00	0.32	0.75
	AsI-BB	14.35	39.00	16.13	27.50	28.00	-0.32	0.75
	RA-UP-R	10.04	7.81	6.12	2.85	29.00	-0.21	0.83
	RA-UP-L	7.25	4.86	6.22	3.45	31.00	0.00	1.00
	RA-lo-R	4.89	3.60	3.44	1.77	29.00	-0.21	0.83
	RA-lo-L	8.37	8.21	2.93	1.25	16.00	-1.59	0.11
	RA-up-tot	13.67	9.57	9.09	3.29	31.00	0.00	1.00
	RA-lo-tot	9.08	6.69	4.85	2.04	23.00	-0.85	0.40
	RA-up-R-%	31.98	16.25	30.41	12.62	28.00	0.32	0.75
	RA-up-L-%	24.53	9.01	30.26	14.05	18.00	1.38	0.17
	RA-lo-R-%	17.23	9.11	17.21	7.74	24.00	0.74	0.46
	RA-lo-L-%	26.26	17.90	13.77	7.15	19.00	-1.27	0.20
	AsI-RA-up	9.01	24.74	2.69	30.14	24.00	-0.74	0.46
	AsI-RA-lo	-14.28	31.82	14.31	23.15	15.00	1.69	0.09
	AEO-R	7.26	11.07	3.33	1.30	26.00	-0.53	0.60
	AEO-L	3.90	1.22	3.75	1.45	31.00	0.00	1.00
	AEO-tot	5.58	5.82	3.40	1.51	27.00	-0.42	0.67
	AEO-R-%	53.06	15.34	42.87	14.53	24.00	-0.74	0.46
	AEO-L-%	46.94	15.34	47.93	14.08	24.00	0.74	0.46
	AsI-AEO	6.12	30.68	-0.64	16.86	24.00	-0.74	0.46
**Standing Position**	BB-R	2.29	0.52	2.21	0.67	27.00	0.42	0.67
	BB-L	2.00	1.55	1.24	0.53	23.00	-0.85	0.40
	BB-tot	2.14	0.70	1.72	0.55	28.50	-0.26	0.79
	BB-R-%	57.28	16.04	58.70	16.83	25.00	0.64	0.53
	BB-L-%	42.72	16.04	32.79	10.73	25.00	-0.64	0.53
	AsI-BB	14.57	32.09	27.06	13.50	25.00	0.64	0.53
	RA-up-R	7.92	4.06	5.83	2.42	24.00	-0.74	0.46
	RA-up-L	6.90	2.61	6.40	2.88	30.00	0.11	0.92
	RA-lo-R	4.23	0.99	4.30	1.90	31.00	0.00	1.00
	RA-lo-L	5.16	3.81	3.20	2.58	13.00	-1.91	0.06
	RA-up-tot	11.37	5.00	9.04	3.83	25.00	-0.64	0.53
	RA-lo-tot	6.81	2.51	5.89	3.01	24.00	-0.74	0.46
	RA-up-R-%	31.74	10.63	27.34	8.57	29.00	0.21	0.83
	RA-up-L-%	28.75	7.99	29.91	9.91	20.00	1.16	0.24
	RA-lo-R-%	18.38	5.66	20.85	7.96	19.00	1.27	0.20
	RA-lo-L-%	21.13	12.50	13.48	5.94	23.00	-0.85	0.40
	AsI-RA-up	4.12	17.55	-2.21	8.42	19.00	-1.27	0.20
	AsI-RA-lo	-1.04	25.19	21.07	18.92	14.00	1.80	0.07

	AEO-R	9.46	6.28	5.34	2.94	18.00	-1.38	0.17
	AEO-L	8.79	6.63	5.29	3.32	19.00	-1.27	0.20
	AEO-tot	9.12	6.27	5.20	2.66	17.00	-1.48	0.14
	AEO-R-%	51.95	8.34	45.24	15.71	19.00	-1.27	0.20
	AEO-L-%	48.05	8.34	47.06	15.82	19.00	1.27	0.20
	AsI-AEO	3.91	16.68	1.82	25.08	19.00	-1.27	0.20
**Sitting Position**	BB-R	2.21	0.70	2.09	0.71	31.00	0.00	1.00
	BB-L	1.87	0.98	1.32	0.86	21.00	-1.06	0.29
	BB-tot	2.04	0.47	1.69	0.69	22.50	-0.90	0.37
	BB-R-%	55.73	16.40	57.31	17.47	22.00	0.95	0.34
	BB-L-%	44.27	16.40	34.38	12.88	22.00	-0.95	0.34
	AsI-BB	11.46	32.79	24.11	19.00	22.00	0.95	0.34
	RA-up-P-R	7.30	3.65	5.17	2.07	23.00	-0.85	0.40
	RA-up-L	7.04	4.80	6.90	4.22	28.00	0.32	0.75
	RA-lo-R	4.76	3.99	3.00	1.22	30.50	-0.05	0.96
	RA-lo-L	5.58	6.88	2.26	0.51	31.00	0.00	1.00
	RA-up-tot	10.82	5.97	8.62	3.81	31.00	0.00	1.00
	RA-lo-tot	7.55	6.83	4.00	1.66	27.00	-0.42	0.67
	RA-up-R-%	32.22	11.07	27.32	9.02	19.00	-1.27	0.20
	RA-up-L-%	29.24	10.50	34.54	14.61	16.00	1.59	0.11
	RA-lo-R-%	19.08	8.43	17.59	8.08	31.00	0.00	1.00
	RA-lo-L-%	19.47	15.28	12.07	5.03	28.00	-0.32	0.75
	ASI-RA-up	4.78	12.06	-7.29	18.22	13.00	-1.91	0.06
	ASI-RA-lo	7.81	28.43	17.04	10.02	25.00	0.64	0.53
	AEO-R	38.72	107.36	2.65	0.94	31.00	0.00	1.00
	AEO-L	3.58	1.20	4.27	2.59	24.00	0.74	0.46
	AEO-tot	21.15	53.87	3.45	1.70	29.00	0.21	0.83
	AEO-R-%	52.13	18.03	37.05	11.13	12.00	-2.01	0.04*
	AEO-L-%	47.87	18.03	54.85	15.04	12.00	2.01	0.04*
	AsI-AEO	4.27	36.06	-14.70	18.17	12.00	-2.01	0.04*
	BB-R	558.33	351.20	683.55	341.43	23.00	0.85	0.40
**Maximum Voluntary Contraction**	BB-L	695.78	357.44	805.66	390.91	22.00	0.95	0.34
	BB-tot	627.06	297.76	738.77	313.15	24.00	0.74	0.46
	BB-R-%	43.45	13.26	41.27	15.54	31.00	0.00	1.00
	BB-L-%	56.55	13.26	50.83	17.81	31.00	0.00	1.00
	AsI-BB	-13.11	26.51	-6.06	27.98	31.00	0.00	1.00
	RA-up-R	88.09	60.46	92.34	46.92	25.00	0.64	0.53
	RA-up-L	97.88	60.37	114.70	77.57	28.00	0.32	0.75
	RA-lo-R	68.36	63.25	109.39	74.80	20.00	1.16	0.24
	RA-lo-L	94.86	147.01	105.75	61.83	13.00	1.91	0.06
	RA-up-tot	137.03	88.76	149.09	71.81	26.00	0.53	0.60
	RA-lo-tot	115.79	135.53	161.56	100.98	18.00	1.38	0.17
	RA-up-R-%	28.45	7.24	20.97	8.07	17.00	-1.48	0.14
	RA-up-L-%	30.28	8.60	25.00	12.35	26.00	-0.53	0.60
	RA-lo-R-%	19.64	4.53	22.49	9.52	23.00	0.85	0.40
	RA-lo-L-%	21.64	8.55	23.46	9.10	15.00	1.69	0.09
	AsI-RA-up	-2.67	19.11	-3.12	22.89	30.00	0.11	0.92
	AsI-RA-lo	-3.16	14.82	-1.03	21.92	29.00	-0.21	0.83

AEO-R	55.77	42.90	98.39	107.79	25.00	0.64	0.53
AEO-L	70.19	48.61	110.64	91.08	24.50	0.69	0.49
AEO-tot	62.98	45.51	103.01	97.40	25.00	0.64	0.53
AEO-R-%	43.00	4.49	39.49	11.83	30.00	0.11	0.92
AEO-L-%	57.00	4.49	52.99	14.61	30.00	-0.11	0.92
AsI-AEO	-14.00	8.98	-9.03	22.41	30.00	0.11	0.92
**FCoI-BB-R**	157.82	126.51	218.48	155.73	20.00	1.16	0.24
**FCoI-BB-L**	360.44	334.80	351.88	219.27	27.00	0.42	0.67
**FCoI-RA-up-R**	16.30	20.08	14.87	5.37	22.00	0.95	0.34
**FCoI-RA-up-L**	17.95	16.80	20.35	14.61	30.00	0.11	0.92
**FCoI-RA-lo-R**	22.12	28.35	32.12	23.30	22.00	0.95	0.34
**FCoI-RA-lo-L**	35.50	84.69	47.09	33.40	11.00	2.12	0.03*
**FCoI-AEO-R**	13.15	11.52	33.29	40.94	19.00	1.27	0.20
**FCoI-AEO-L**	18.96	12.87	35.63	34.82	27.00	0.42	0.67
**FCoSI-BB**	-27.06	31.60	-15.98	33.77	27.00	0.42	0.67
**FCoSI-RA-up**	-11.80	24.82	-1.61	30.77	28.00	0.32	0.75
**FCoSI-RA-lo**	11.36	37.49	-9.92	32.49	18.00	-1.38	0.17
**FCoSI-AEO**	-18.90	29.90	-3.15	29.13	27.00	0.42	0.67

AcI— activity index; AEO— abdominal external oblique muscle; AsI— asymmetry index; BB — biceps brachii muscle; ES—effect size; FCAI— Functional Clenching Activity Index; FCI — Functional Clenching Index; FCoI— Functional Contraction Index; FCoSI— Functional Contraction Symmetry Index; FCSI— Functional Clenching Symmetry Index; L— left side; MM — the superficial part of the masseter muscle; MVC— maximum voluntary contraction; n— individuals in the sample; POC— percentage overlapping coefficient; R— right side; RA-lo — the lower part of the rectus abdominis muscle; RA-up— the upper part of the rectus abdominis muscle; SCM — the middle part of the sternocleidomastoid muscle; SD — standard deviation; TA — the anterior part of the temporalis muscle; TC— torque coefficient; U— the difference between the two rank totals; UT— the upper part of the trapezius muscle; Z— the Mann-Whitney U test; * significant difference.

Table S9. Comparison of bioelectrical activity findings of postural muscles and the arm muscles in emmetropic subjects and myopic subjects (Accommodation Paralysis in The Left Eye).

		**Emmetropic Subjects**		**Myopic Subjects**		**U**	**Z**	**P**
		Mean	SD	Mean	SD			
**Lying Position**	BB-R	4.99	4.45	2.87	1.14	21.00	-1.06	0.29
	BB-L	3.75	3.77	2.21	1.18	29.00	-0.21	0.83
	BB-tot	4.37	3.58	2.52	0.90	21.00	-1.06	0.29
	BB-R-%	61.92	15.81	56.10	16.61	25.00	-0.64	0.53
	BB-L-%	38.08	15.81	35.69	10.32	25.00	0.64	0.53
	AsI-BB	23.85	31.62	23.56	13.22	25.00	-0.64	0.53
	RA-up-R	10.70	8.36	5.76	2.89	22.00	-0.95	0.34
	RA-up-L	12.90	12.52	5.72	2.85	27.00	-0.42	0.67
	RA-lo-R	5.47	3.94	4.52	4.29	28.00	-0.32	0.75
	RA-lo-L	7.53	9.08	2.73	1.58	21.00	-1.06	0.29
	RA-up-tot	17.15	14.41	8.46	3.46	21.00	-1.06	0.29
	RA-lo-tot	9.24	7.64	5.84	4.74	26.00	-0.53	0.60
	RA-up-R-%	30.01	6.52	29.55	12.94	26.00	0.53	0.60
	RA-up-L-%	33.36	13.79	29.08	12.38	28.00	-0.32	0.75
	RA-lo-R-%	17.70	10.10	20.69	12.67	22.00	0.95	0.34
	RA-lo-L-%	18.92	11.83	13.38	4.71	25.00	-0.64	0.53
	AsI-RA-up	-1.55	18.48	4.43	28.18	31.00	0.00	1.00
	AsI-RA-lo	-1.52	34.61	19.25	21.37	22.00	0.95	0.34
	AEO-R	6.79	8.96	3.29	1.85	24.00	-0.74	0.46
	AEO-L	7.54	11.06	3.48	1.76	26.50	-0.48	0.63
	AEO-tot	7.17	6.68	3.40	1.76	22.00	-0.95	0.34
	AEO-R-%	49.90	21.95	44.29	12.87	27.00	-0.42	0.67
	AEO-L-%	50.10	21.95	46.79	13.99	27.00	0.42	0.67
	AsI-AEO	-0.21	43.90	-0.45	11.53	27.00	-0.42	0.67
**Standing Position**	BB-R	2.59	1.22	2.46	1.58	31.00	0.00	1.00
	BB-L	1.55	0.56	2.14	3.01	23.00	-0.85	0.40
	BB-tot	2.07	0.58	2.29	2.27	19.00	-1.27	0.20
	BB-R-%	61.28	13.58	55.33	18.72	29.00	0.21	0.83
	BB-L-%	38.72	13.58	35.61	14.47	29.00	-0.21	0.83
	AsI-BB	22.55	27.17	20.65	21.01	29.00	0.21	0.83
	RA-up-R	8.25	5.25	6.40	2.72	30.00	0.11	0.92
	RA-up-L	8.88	5.00	6.70	2.79	29.00	-0.21	0.83
	RA-lo-R	3.90	0.78	6.69	4.07	13.00	1.91	0.06
	RA-lo-L	3.05	1.06	5.05	3.54	25.00	0.64	0.53
	RA-up-tot	12.70	6.79	9.75	4.06	29.00	-0.21	0.83
	RA-lo-tot	5.42	1.10	9.19	5.30	17.00	1.48	0.14
	RA-up-R-%	32.76	10.20	24.47	8.80	18.00	-1.38	0.17
	RA-up-L-%	35.57	12.23	25.30	9.02	18.00	-1.38	0.17
	RA-lo-R-%	17.60	5.29	24.70	10.87	11.00	2.12	0.03*
	RA-lo-L-%	14.06	6.63	17.08	9.44	22.00	0.95	0.34
	AsI-RA-up	-3.07	27.60	-0.16	8.42	19.00	-1.27	0.20
	AsI-RA-lo	13.52	15.44	19.95	22.72	24.00	0.74	0.46

	AEO-R	8.92	5.61	5.31	2.12	20.00	-1.16	0.24
	AEO-L	7.62	4.48	5.41	2.37	21.00	-1.06	0.29
	AEO-tot	8.27	4.70	5.30	1.88	21.00	-1.06	0.29
	AEO-R-%	52.48	10.24	45.69	15.24	27.00	-0.42	0.67
	AEO-L-%	47.52	10.24	45.75	15.22	27.00	0.42	0.67
	AsI-AEO	4.96	20.47	3.09	20.52	27.00	-0.42	0.67
**Sitting Position**	BB-R	2.39	0.66	1.95	0.68	23.50	-0.79	0.43
	BB-L	1.61	0.61	1.32	0.55	27.00	-0.42	0.67
	BB-tot	2.00	0.46	1.63	0.54	20.00	-1.16	0.24
	BB-R-%	60.14	10.35	55.61	17.18	31.00	0.00	1.00
	BB-L-%	39.86	10.35	35.69	11.95	31.00	0.00	1.00
	AsI-BB	20.27	20.69	21.72	15.24	31.00	0.00	1.00
	RA-up-P-R	6.90	3.06	5.29	2.43	25.00	-0.64	0.53
	RA-up-L	6.10	5.35	8.70	8.87	21.00	1.06	0.29
	RA-lo-R	3.71	1.60	3.42	1.97	31.00	0.00	1.00
	RA-lo-L	5.44	7.25	1.92	1.01	27.00	-0.42	0.67
	RA-up-tot	9.96	5.41	9.60	5.33	27.00	0.42	0.67
	RA-lo-tot	6.43	5.07	4.41	2.38	30.00	-0.11	0.92
	RA-up-R-%	32.80	8.42	27.42	12.76	26.00	-0.53	0.60
	RA-up-L-%	28.44	16.50	35.44	18.35	24.00	0.74	0.46
	RA-lo-R-%	17.66	4.71	18.84	10.53	29.00	0.21	0.83
	RA-lo-L-%	21.10	20.21	10.40	4.38	24.00	-0.74	0.46
	AsI-RA-up	16.67	35.72	-6.44	30.53	18.00	-1.38	0.17
	AsI-RA-lo	6.89	34.76	24.58	15.97	23.00	0.85	0.40
	AEO-R	2.67	0.56	2.74	0.98	23.50	0.79	0.43
	AEO-L	3.48	0.95	3.95	1.52	23.00	0.85	0.40
	AEO-tot	3.07	0.70	3.34	1.18	25.00	0.64	0.53
	AEO-R-%	43.79	4.83	38.75	12.11	30.00	0.11	0.92
	AEO-L-%	56.21	4.83	52.02	15.66	30.00	-0.11	0.92
	AsI-AEO	-12.42	9.66	-10.51	14.91	30.00	0.11	0.92
	BB-R	662.72	399.49	834.93	348.90	19.00	1.27	0.20
**Maximum Voluntary Contraction**	BB-L	776.61	417.01	1355.43	1546.21	21.00	1.06	0.29
	BB-tot	719.67	364.53	1088.55	794.15	18.00	1.38	0.17
	BB-R-%	45.34	11.75	41.60	18.45	28.00	0.32	0.75
	BB-L-%	54.66	11.75	50.07	19.98	28.00	-0.32	0.75
	AsI-BB	-9.32	23.50	-4.68	33.05	28.00	0.32	0.75
	RA-up-R	91.94	51.88	116.75	63.47	19.00	1.27	0.20
	RA-up-L	110.13	73.91	126.57	49.71	24.00	0.74	0.46
	RA-lo-R	77.37	84.26	156.22	121.83	17.00	1.48	0.14
	RA-lo-L	107.68	182.27	139.09	98.93	13.00	1.91	0.06
	RA-up-tot	147.00	87.08	178.30	86.65	20.00	1.16	0.24
	RA-lo-tot	131.21	174.84	225.17	169.88	16.00	1.59	0.11
	RA-up-R-%	28.35	8.90	21.24	6.99	18.00	-1.38	0.17
	RA-up-L-%	31.68	7.95	24.36	8.53	21.00	-1.06	0.29
	RA-lo-R-%	19.44	4.20	23.62	9.07	17.00	1.48	0.14
	RA-lo-L-%	20.52	10.21	23.16	8.19	16.00	1.59	0.11
	AsI-RA-up	-6.37	18.05	-1.44	16.33	29.00	0.21	0.83
	AsI-RA-lo	0.51	15.72	3.17	19.44	30.00	-0.11	0.92

AEO-R	79.87	76.96	123.96	114.30	23.00	0.85	0.40
AEO-L	131.52	205.10	134.00	83.44	21.00	1.06	0.29
AEO-tot	105.70	138.76	128.73	92.82	22.00	0.95	0.34
AEO-R-%	45.44	9.78	40.92	16.30	31.00	0.00	1.00
AEO-L-%	54.56	9.78	49.88	18.18	31.00	0.00	1.00
AsI-AEO	-9.13	19.56	-5.81	25.31	31.00	0.00	1.00
**FCoI-BB-R**	181.54	148.94	305.95	153.26	14.00	1.80	0.07
**FCoI-BB-L**	418.28	425.19	796.52	945.94	15.00	1.69	0.09
**FCoI-RA-up-R**	13.77	14.35	20.31	13.37	14.00	1.80	0.07
**FCoI-RA-up-L**	17.53	19.71	25.24	13.86	18.00	1.38	0.17
**FCoI-RA-lo-R**	24.38	39.19	39.51	29.38	14.00	1.80	0.07
**FCoI-RA-lo-L**	43.88	102.44	56.61	40.90	7.00	2.54	0.01*
**FCoI-AEO-R**	16.64	16.00	46.35	52.45	22.00	0.95	0.34
**FCoI-AEO-L**	38.10	73.02	47.25	43.36	17.00	1.48	0.14
**FCoSI-BB**	-30.30	35.56	-22.26	36.80	30.00	0.11	0.92
**FCoSI-RA-up**	-4.61	22.95	-1.13	27.12	30.00	-0.11	0.92
**FCoSI-RA-lo**	2.15	34.22	-12.03	28.00	20.00	-1.16	0.24
**FCoSI-AEO**	-6.54	45.45	-2.01	31.99	29.00	0.21	0.83

AcI— activity index; AEO— abdominal external oblique muscle; ASI— asymmetry index; BB — biceps brachii muscle; ES—effect size; FCAI— Functional Clenching Activity Index; FCI — Functional Clenching Index;; FCoI— Functional Contraction Index; FCoSI— Functional Contraction Symmetry Index; FCSI— Functional Clenching Symmetry Index; L— left side; MM — the superficial part of the masseter muscle; MVC— maximum voluntary contraction; n— individuals in the sample; POC— percentage overlapping coefficient; R— right side; RA-lo — the lower part of the rectus abdominis muscle; RA-up— the upper part of the rectus abdominis muscle; SCM — the middle part of the sternocleidomastoid muscle; SD — standard deviation; TA — the anterior part of the temporalis muscle; TC— torque coefficient; U— the difference between the two rank totals; UT— the upper part of the trapezius muscle; Z— the Mann-Whitney U test; * significant difference.

Table S10. Comparison of bioelectrical activity findings of postural muscles and the arm muscles in emmetropic subjects.

		Without Paralysis of Accommodation		Accommodation Paralysis in The Right Eye		Accommodation Paralysis in The Left Eye		H	P
		Mean	SD	Mean	SD	Mean	SD		
Lying Position	BB-R	4.11	2.00	3.80	1.36	4.99	4.45	0.014	0.99
	BB-L	4.68	6.52	4.45	5.01	3.75	3.77	0.03	0.98
	BB-tot	4.39	3.90	4.12	2.97	4.37	3.58	0.014	0.99
	BB-R-%	58.86	18.25	57.18	19.50	61.92	15.81	0.32	0.85
	BB-L-%	41.14	18.25	42.82	19.50	38.08	15.81	0.32	0.85
	AsI-BB	17.71	36.49	14.35	39.00	23.85	31.62	0.32	0.85
	RA-up-R	7.38	3.21	10.04	7.81	10.70	8.36	0.39	0.82
	RA-up-L	6.52	3.26	7.25	4.86	12.90	12.52	0.68	0.71
	RA-lo-R	4.91	1.73	4.89	3.60	5.47	3.94	0.66	0.71
	RA-lo-L	10.81	9.67	8.37	8.21	7.53	9.08	1.08	0.58
	RA-up-tot	10.64	4.48	13.67	9.57	17.15	14.41	0.4	0.82
	RA-lo-tot	10.32	5.19	9.08	6.69	9.24	7.64	0.13	0.93
	RA-up-R-%	26.61	9.07	31.98	16.25	30.01	6.52	0.05	0.97
	RA-up-L-%	22.99	7.58	24.53	9.01	33.36	13.79	0.24	0.88
	RA-lo-R-%	18.73	7.96	17.23	9.11	17.70	10.10	3.27	0.19
	RA-lo-L-%	31.67	18.98	26.26	17.90	18.92	11.83	0.24	0.88
	AsI-RA-up	6.18	19.03	9.01	24.74	-1.55	18.48	1.03	0.59
	AsI-RA-lo	-17.69	40.54	-14.28	31.82	-1.52	34.61	0.96	0.62
	AEO-R	6.71	5.16	7.26	11.07	6.79	8.96	1.29	0.52
	AEO-L	5.24	4.22	3.90	1.22	7.54	11.06	0.03	0.98
	AEO-tot	5.97	4.59	5.58	5.82	7.17	6.68	0.55	0.75
	AEO-R-%	55.24	11.06	53.06	15.34	49.90	21.95	0.68	0.71
	AEO-L-%	44.76	11.06	46.94	15.34	50.10	21.95	0.68	0.71
	AsI-AEO	10.48	22.12	6.12	30.68	-0.21	43.90	0.67	0.71
Standing Position	BB-R	2.45	0.71	2.29	0.52	2.59	1.22	0.1	0.95
	BB-L	1.77	0.98	2.00	1.55	1.55	0.56	0.24	0.88
	BB-tot	2.11	0.62	2.14	0.70	2.07	0.58	0.03	0.98
	BB-R-%	59.10	12.02	57.28	16.04	61.28	13.58	0.55	0.75
	BB-L-%	40.90	12.02	42.72	16.04	38.72	13.58	0.55	0.75
	AsI-BB	18.20	24.05	14.57	32.09	22.55	27.17	0.55	0.75
	RA-up-R	8.72	6.10	7.92	4.06	8.25	5.25	0.1	0.95
	RA-up-L	7.46	3.85	6.90	2.61	8.88	5.00	0.23	0.89
	RA-lo-R	4.14	0.59	4.23	0.99	3.90	0.78	0.65	0.72
	RA-lo-L	5.81	8.47	5.16	3.81	3.05	1.06	1.78	0.4
	RA-up-tot	12.44	7.92	11.37	5.00	12.70	6.79	0.09	0.95
	RA-lo-tot	7.05	4.58	6.81	2.51	5.42	1.10	1.19	0.55

	RA-up-R-%	32.73	11.05	31.74	10.63	32.76	10.20	0.22	0.89
	RA-up-L-%	29.52	7.00	28.75	7.99	35.57	12.23	1.45	0.48
	RA-lo-R-%	18.55	7.42	18.38	5.66	17.60	5.29	0.05	0.97
	RA-lo-L-%	19.20	15.99	21.13	12.50	14.06	6.63	1.81	0.4
	ASI-RA-up	3.71	15.15	4.12	17.55	-3.07	27.60	0	0.99
	ASI-RA-lo	6.00	32.53	-1.04	25.19	13.52	15.44	2.01	0.36
	AEO-R	9.04	5.57	9.46	6.28	8.92	5.61	0.05	0.97
	AEO-L	7.59	3.95	8.79	6.63	7.62	4.48	0.05	0.97
	AEO-tot	8.31	4.54	9.12	6.27	8.27	4.70	0.07	0.96
	AEO-R-%	52.53	8.91	51.95	8.34	52.48	10.24	0.32	0.85
	AEO-L-%	47.47	8.91	48.05	8.34	47.52	10.24	0.32	0.85
	AsI-AEO	5.05	17.83	3.91	16.68	4.96	20.47	0.32	0.85
	BB-R	2.23	0.69	2.21	0.70	2.39	0.66	0.32	0.85
	BB-L	1.46	0.89	1.87	0.98	1.61	0.61	1.37	0.5
	BB-tot	1.84	0.56	2.04	0.47	2.00	0.46	1.17	0.55
	BB-R-%	61.52	13.33	55.73	16.40	60.14	10.35	0.53	0.76
	BB-L-%	38.48	13.33	44.27	16.40	39.86	10.35	0.53	0.76
	AsI-BB	23.04	26.65	11.46	32.79	20.27	20.69	0.53	0.76
	RA-up-P-R	6.60	4.20	7.30	3.65	6.90	3.06	1.12	0.57
	RA-up-L	7.72	7.26	7.04	4.80	6.10	5.35	0.6	0.73
	RA-lo-R	3.73	1.16	4.76	3.99	3.71	1.60	0.14	0.93
	RA-lo-L	6.18	11.77	5.58	6.88	5.44	7.25	0.53	0.76
	RA-up-tot	10.46	5.66	10.82	5.97	9.96	5.41	0.15	0.92
	RA-lo-tot	6.81	6.40	7.55	6.83	6.43	5.07	0.26	0.87
**Sitting Position**	RA-up-R-%	32.28	13.45	32.22	11.07	32.80	8.42	0.14	0.93
	RA-up-L-%	30.79	5.43	29.24	10.50	28.44	16.50	0.17	0.91
	RA-lo-R-%	19.38	8.05	19.08	8.43	17.66	4.71	0.45	0.79
	RA-lo-L-%	17.56	13.23	19.47	15.28	21.10	20.21	0.01	0.99
	AsI-RA-up	-1.58	30.28	4.78	12.06	16.67	35.72	0.66	0.71
	AsI-RA-lo	11.18	36.31	7.81	28.43	6.89	34.76	0.14	0.93
	AEO-R	3.14	0.73	38.72	107.36	2.67	0.56	1.67	0.43
	AEO-L	3.63	1.11	3.58	1.20	3.48	0.95	0.09	0.95
	AEO-tot	3.39	0.83	21.15	53.87	3.07	0.70	0.65	0.72
	AEO-R-%	46.86	6.42	52.13	18.03	43.79	4.83	2.51	0.28
	AEO-L-%	53.14	6.42	47.87	18.03	56.21	4.83	2.51	0.28
	AsI-AEO	-6.28	12.83	4.27	36.06	-12.42	9.66	2.51	0.28
	BB-R	477.53	359.67	558.33	351.20	662.72	399.49	1.07	0.58
	BB-L	618.89	390.92	695.78	357.44	776.61	417.01	1.15	0.56

**Maximum Voluntary Contraction**	BB-tot	548.21	329.99	627.06	297.76	719.67	364.53	1.02	0.6
	BB-R-%	41.85	12.78	43.45	13.26	45.34	11.75	0.65	0.72
	BB-L-%	58.15	12.78	56.55	13.26	54.66	11.75	0.65	0.72
	AsI-BB	-16.31	25.56	-13.11	26.51	-9.32	23.50	0.65	0.72
	RA-up-R	97.55	78.76	88.09	60.46	91.94	51.88	0.24	0.88
	RA-up-L	109.19	80.82	97.88	60.37	110.13	73.91	0.03	0.98
	RA-lo-R	69.26	72.09	68.36	63.25	77.37	84.26	0.07	0.961
	RA-lo-L	300.63	601.07	94.86	147.01	107.68	182.27	0.05	0.97
	RA-up-tot	152.14	117.79	137.03	88.76	147.00	87.08	0.15	0.92
	RA-lo-tot	219.57	315.55	115.79	135.53	131.21	174.84	0.14	0.93
	RA-up-R-%	26.52	12.06	28.45	7.24	28.35	8.90	0.03	0.98
	RA-up-L-%	28.36	11.14	30.28	8.60	31.68	7.95	0.22	0.89
	RA-lo-R-%	17.09	7.28	19.64	4.53	19.44	4.20	0.38	0.82
	RA-lo-L-%	28.03	26.27	21.64	8.55	20.52	10.21	0.29	0.86
	ASI-RA-up	-6.91	13.62	-2.67	19.11	-6.37	18.05	0.12	0.94
	ASI-RA-lo	-8.92	37.90	-3.16	14.82	0.51	15.72	0.65	0.72
	AEO-R	65.49	71.55	55.77	42.90	79.87	76.96	0.91	0.63
	AEO-L	87.22	114.21	70.19	48.61	131.52	205.10	0.23	0.89
	AEO-tot	76.35	92.78	62.98	45.51	105.70	138.76	0.45	0.79
	AEO-R-%	46.62	5.27	43.00	4.49	45.44	9.78	2.95	0.22
	AEO-L-%	53.38	5.27	57.00	4.49	54.56	9.78	2.95	0.22
	AsI-AEO	-6.76	10.54	-14.00	8.98	-9.13	19.56	2.95	0.22
**FCoI-BB-R**		127.63	107.12	157.82	126.51	181.54	148.94	0.53	0.76
**FCoI-BB-L**		321.70	337.60	360.44	334.80	418.28	425.19	0.13	0.93
**FCoI-RA-up-R**		15.24	14.80	16.30	20.08	13.77	14.35	0.09	0.95
**FCoI-RA-up-L**		19.83	18.39	17.95	16.80	17.53	19.71	0.39	0.82
**FCoI-RA-lo-R**		18.06	26.95	22.12	28.35	24.38	39.19	0.13	0.93
**FCoI-RA-lo-L**		83.55	164.86	35.50	84.69	43.88	102.44	1.08	0.58
**FCoI-AEO-R**		14.77	17.92	13.15	11.52	16.64	16.00	0.86	0.65
**FCoI-AEO-L**		28.13	43.03	18.96	12.87	38.10	73.02	0.39	0.82
**FCoSI-BB**		-32.27	36.44	-27.06	31.60	-30.30	35.56	0.26	0.89
**FCoSI-RA-up**		-13.02	19.41	-11.80	24.82	-4.61	22.95	0.88	0.64
**FCoSI-RA-lo**		9.19	57.75	11.36	37.49	2.15	34.22	0.52	0.77
**FCoSI-AEO**		-16.18	28.00	-18.90	29.90	-6.54	45.45	0.95	0.62

AcI— activity index; AEO— abdominal external oblique muscle; AsI— asymmetry index; BB — biceps brachii muscle; ES—effect size; FCAI— Functional Clenching Activity Index; FCI — Functional Clenching Index; FCoI— Functional Contraction Index; FCoSI— Functional Contraction Symmetry Index; FCSI— Functional Clenching Symmetry Index; L— left side; MM — the superficial part of the masseter muscle; MVC— maximum voluntary contraction; n— individuals in the sample; POC— percentage overlapping coefficient; R— right side; RA-lo — the lower part of the rectus abdominis muscle; RA-up— the upper part of the rectus abdominis muscle; SCM — the middle part of the sternocleidomastoid muscle; SD — standard deviation; TA — the anterior part of the temporalis muscle; TC— torque coefficient; U— the difference between the two rank totals; UT— the upper part of the trapezius muscle; H— the Kruskal-Wallis test.

Table S11. Comparison of bioelectrical activity findings of postural muscles and the arm muscles in myopic subjects.

		**Without Paralysis of Accommodation**		**Accommodation Paralysis in The Right Eye**		**Accommodation Paralysis in The Left Eye**		**H**	**P**
		Mean	SD	Mean	SD	Mean	SD		
**Lying Position**	BB-R	3.76	2.38	3.68	2.02	2.87	1.14	0.68	0.71
	BB-L	2.78	1.37	3.52	4.23	2.21	1.18	1.12	0.57
	BB-tot	3.20	1.56	3.56	2.95	2.52	0.90	1.09	0.57
	BB-R-%	51.87	21.41	52.49	18.34	56.10	16.61	0.14	0.93
	BB-L-%	39.58	18.39	39.98	15.96	35.69	10.32	0.14	0.93
	AsI-BB	16.24	31.98	16.13	27.50	23.56	13.22	0.14	0.93
	RA-up-R	7.24	3.16	6.12	2.85	5.76	2.89	1.14	0.56
	RA-up-L	6.31	2.75	6.22	3.45	5.72	2.85	0.45	0.79
	RA-lo-R	3.52	0.87	3.44	1.77	4.52	4.29	0.05	0.97
	RA-lo-L	4.52	2.47	2.93	1.25	2.73	1.58	1.52	0.46
	RA-up-tot	10.37	3.42	9.09	3.29	8.46	3.46	0.72	0.69
	RA-lo-tot	5.68	1.67	4.85	2.04	5.84	4.74	0.14	0.93
	RA-up-R-%	31.19	13.44	30.41	12.62	29.55	12.94	0.45	0.79
	RA-up-L-%	26.73	9.01	30.26	14.05	29.08	12.38	0.21	0.9
	RA-lo-R-%	17.03	5.45	17.21	7.74	20.69	12.67	0.36	0.83
	RA-lo-L-%	17.83	5.30	13.77	7.15	13.38	4.71	2.46	0.29
	AsI-RA-up	10.17	24.80	2.69	30.14	4.43	28.18	0.05	0.97
	AsI-RA-lo	2.74	25.31	14.31	23.15	19.25	21.37	3.57	0.16
	AEO-R	3.46	2.30	3.33	1.30	3.29	1.85	1.12	0.57
	AEO-L	5.35	2.83	3.75	1.45	3.48	1.76	0.04	0.98
	AEO-tot	3.99	1.42	3.40	1.51	3.40	1.76	0.04	0.98
	AEO-R-%	36.17	10.05	42.87	14.53	44.29	12.87	6.7	0.03
	AEO-L-%	56.82	16.33	47.93	14.08	46.79	13.99	6.7	0.03
	AsI-AEO	-15.16	29.01	-0.64	16.86	-0.45	11.53	6.7	0.03
**Standing Position**	BB-R	2.23	0.85	2.21	0.67	2.46	1.58	0.44	0.79
	BB-L	1.14	0.37	1.24	0.53	2.14	3.01	0.84	0.65
	BB-tot	1.65	0.57	1.72	0.55	2.29	2.27	0.84	0.65
	BB-R-%	58.70	20.27	58.70	16.83	55.33	18.72	0.29	0.86
	BB-L-%	32.20	11.63	32.79	10.73	35.61	14.47	0.29	0.86
	AsI-BB	29.90	17.77	27.06	13.50	20.65	21.01	0.29	0.86
	RA-up-R	7.07	3.51	5.83	2.42	6.40	2.72	0.36	0.83
	RA-up-L	7.17	3.22	6.40	2.88	6.70	2.79	0.43	0.8
	RA-lo-R	4.80	2.63	4.30	1.90	6.69	4.07	2.03	0.36
	RA-lo-L	3.69	2.75	3.20	2.58	5.05	3.54	2.1	0.34
	RA-up-tot	10.54	5.16	9.04	3.83	9.75	4.06	0.36	0.83
	RA-lo-tot	6.62	3.91	5.89	3.01	9.19	5.30	2.27	0.32

	RA-up-R-%	27.25	8.41	27.34	8.57	24.47	8.80	1.66	0.43
	RA-up-L-%	28.50	8.70	29.91	9.91	25.30	9.02	2.83	0.24
	RA-lo-R-%	20.85	9.36	20.85	7.96	24.70	10.87	0.94	0.62
	RA-lo-L-%	15.20	6.22	13.48	5.94	17.08	9.44	0.83	0.66
	ASI-RA-up	2.05	13.31	-2.21	8.42	-0.16	8.42	1.61	0.44
	ASI-RA-lo	16.68	16.75	21.07	18.92	19.95	22.72	1.19	0.91
	AEO-R	5.89	4.27	5.34	2.94	5.31	2.12	0.95	0.62
	AEO-L	5.82	2.79	5.29	3.32	5.41	2.37	0.45	0.79
	AEO-tot	5.81	2.65	5.20	2.66	5.30	1.88	0.23	0.89
	AEO-R-%	42.66	18.50	45.24	15.71	45.69	15.24	0.39	0.82
	AEO-L-%	47.40	19.90	47.06	15.82	45.75	15.22	0.39	0.82
	AsI-AEO	-2.18	27.68	1.82	25.08	3.09	20.52	0.39	0.82
	BB-R	2.09	0.88	2.09	0.71	1.95	0.68	0.27	0.87
	BB-L	1.20	0.49	1.32	0.86	1.32	0.55	1.21	0.54
	BB-tot	1.62	0.63	1.69	0.69	1.63	0.54	0.1	0.95
	BB-R-%	57.27	20.88	57.31	17.47	55.61	17.18	0.45	0.79
	BB-L-%	32.82	12.62	34.38	12.88	35.69	11.95	0.45	0.79
	AsI-BB	27.03	17.71	24.11	19.00	21.72	15.24	0.45	0.79
**Sitting Position**	RA-up-P-R	5.13	2.15	5.17	2.07	5.29	2.43	0	1
	RA-up-L	6.20	2.31	6.90	4.22	8.70	8.87	0.05	0.97
	RA-lo-R	2.93	0.81	3.00	1.22	3.42	1.97	0.16	0.92
	RA-lo-L	2.81	1.88	2.26	0.51	1.92	1.01	0.5	0.77
	RA-up-tot	8.19	3.27	8.62	3.81	9.60	5.33	0.21	0.9
	RA-lo-tot	4.32	0.75	4.00	1.66	4.41	2.38	0.36	0.83
	RA-up-R-%	28.17	9.26	27.32	9.02	27.42	12.76	0.03	0.98
	RA-up-L-%	33.33	8.92	34.54	14.61	35.44	18.35	0.09	0.95
	RA-lo-R-%	17.91	9.16	17.59	8.08	18.84	10.53	0.36	0.83
	RA-lo-L-%	14.31	4.79	12.07	5.03	10.40	4.38	0.69	0.7
	AsI-RA-up	-1.53	16.65	-7.29	18.22	-6.44	30.53	0.47	0.79
	AsI-RA-lo	19.97	17.09	17.04	10.02	24.58	15.97	2.23	0.32
	AEO-R	2.40	1.01	2.65	0.94	2.74	0.98	0.94	0.62
	AEO-L	3.83	1.88	4.27	2.59	3.95	1.52	0.1	0.95
	AEO-tot	3.11	1.34	3.45	1.70	3.34	1.18	0.11	0.94
	AEO-R-%	35.16	14.22	37.05	11.13	38.75	12.11	1.65	0.55
	AEO-L-%	53.55	20.97	54.85	15.04	52.02	15.66	1.17	0.55
	AsI-AEO	-17.19	18.06	-14.70	18.17	-10.51	14.91	1.17	0.55
	BB-R	825.12	471.41	683.55	341.43	834.93	348.90	1.63	0.44
	BB-L	858.94	384.31	805.66	390.91	1355.43	1546.21	0.72	0.69

	BB-tot	836.57	381.18	738.77	313.15	1088.55	794.15	2.15	0.34
	BB-R-%	43.23	17.80	41.27	15.54	41.60	18.45	0.36	0.83
	BB-L-%	47.21	18.98	50.83	17.81	50.07	19.98	0.36	0.83
	AsI-BB	-1.04	26.82	-6.06	27.98	-4.68	33.05	0.36	0.83
	RA-up-R	126.78	66.40	92.34	46.92	116.75	63.47	1.99	0.36
	RA-up-L	136.47	90.76	114.70	77.57	126.57	49.71	1.4	0.49
	RA-lo-R	131.72	95.12	109.39	74.80	156.22	121.83	0.72	0.69
	RA-lo-L	150.06	73.42	105.75	61.83	139.09	98.93	0.74	0.69
	RA-up-tot	194.79	106.93	149.09	71.81	178.30	86.65	1.99	0.36
	RA-lo-tot	206.68	126.19	161.56	100.98	225.17	169.88	0.76	0.68
	RA-up-R-%	22.89	7.23	20.97	8.07	21.24	6.99	0.83	0.66
**Maximum Voluntary Contraction**	RA-up-L-%	24.07	9.94	25.00	12.35	24.36	8.53	0.14	0.93
	RA-lo-R-%	20.41	10.83	22.49	9.52	23.62	9.07	0.83	0.66
	RA-lo-L-%	24.04	7.33	23.46	9.10	23.16	8.19	0.14	0.93
	AsI-RA-up	0.89	14.20	-3.12	22.89	-1.44	16.33	0.36	0.83
	AsI-RA-lo	-6.21	21.45	-1.03	21.92	3.17	19.44	1.17	0.55
	AEO-R	111.93	127.79	98.39	107.79	123.96	114.30	0.68	0.71
	AEO-L	162.56	89.60	110.64	91.08	134.00	83.44	0.76	0.68
	AEO-tot	136.96	96.70	103.01	97.40	128.73	92.82	1.12	0.57
	AEO-R-%	36.43	17.48	39.49	11.83	40.92	16.30	0.42	0.80
	AEO-L-%	53.51	22.40	52.99	14.61	49.88	18.18	0.42	0.80
	AsI-AEO	-14.63	30.82	-9.03	22.41	-5.81	25.31	0.42	0.80
**FCoI-BB-R**		260.56	141.83	218.48	155.73	305.95	153.26	3.44	0.17
**FCoI-BB-L**		404.66	146.74	351.88	219.27	796.52	945.94	3.5	0.17
**FCoI-RA-up-R**		22.04	15.58	14.87	5.37	20.31	13.37	1.7	0.42
**FCoI-RA-up-L**		26.96	19.54	20.35	14.61	25.24	13.86	0.96	0.61
**FCoI-RA-lo-R**		42.40	31.61	32.12	23.30	39.51	29.38	0.68	0.71
**FCoI-RA-lo-L**		60.46	50.35	47.09	33.40	56.61	40.90	0.39	0.82
**FCoI-AEO-R**		39.21	41.32	33.29	40.94	46.35	52.45	1.43	0.48
**FCoI-AEO-L**		40.94	24.59	35.63	34.82	47.25	43.36	0.32	0.85
**FCoSI-BB**		-11.79	35.85	-15.98	33.77	-22.26	36.80	0.2	0.9
**FCoSI-RA-up**		-6.60	25.22	-1.61	30.77	-1.13	27.12	0.14	0.93
**FCoSI-RA-lo**		-2.12	34.13	-9.92	32.49	-12.03	28.00	0.47	0.79
**FCoSI-AEO**		8.97	39.04	-3.15	29.13	-2.01	31.99	1.12	0.57

AcI— activity index; AEO— abdominal external oblique muscle; AsI— asymmetry index; BB — biceps brachii muscle; ES—effect size; FCAI— Functional Clenching Activity Index; FCI — Functional Clenching Index; FCoI—Functional Contraction Index; FCoSI— Functional Contraction Symmetry Index; FCSI— Functional Clenching Symmetry Index; L— left side; MM — the superficial part of the masseter muscle; MVC— maximum voluntary contraction; n— individuals in the sample; POC— percentage overlapping coefficient; R— right side; RA-lo — the lower part of the rectus abdominis muscle; RA-up— the upper part of the rectus abdominis muscle; SCM — the middle part of the sternocleidomastoid muscle; SD — standard deviation; TA — the anterior part of the temporalis muscle; TC— torque coefficient; U— the difference between the two rank totals; UT— the upper part of the trapezius muscle; H— the Kruskal-Wallis test; * significant difference.
